# Lineage tracing of axial progenitors using Nkx1-2CreER^T2^ mice defines their trunk and tail contributions

**DOI:** 10.1242/dev.164319

**Published:** 2018-10-02

**Authors:** Aida Rodrigo Albors, Pamela A. Halley, Kate G. Storey

**Affiliations:** Neural Development Group, Division of Cell and Developmental Biology, School of Life Sciences, University of Dundee, Dow Street, Dundee DD1 5EH, UK

**Keywords:** Axial progenitors, Neuromesodermal progenitors, Nkx1-2, Body axis elongation, Genetic lineage tracing, Mouse embryo

## Abstract

The vertebrate body forms by continuous generation of new tissue from progenitors at the posterior end of the embryo. The study of these axial progenitors has proved to be challenging *in vivo* largely because of the lack of unique molecular markers to identify them. Here, we elucidate the expression pattern of the transcription factor *Nkx1-2* in the mouse embryo and show that it identifies axial progenitors throughout body axis elongation, including neuromesodermal progenitors and early neural and mesodermal progenitors. We create a tamoxifen-inducible Nkx1-2CreER^T2^ transgenic mouse and exploit the conditional nature of this line to uncover the lineage contributions of *Nkx1-2*-expressing cells at specific stages. We show that early *Nkx1-2*-expressing epiblast cells contribute to all three germ layers, mostly neuroectoderm and mesoderm, excluding notochord. Our data are consistent with the presence of some self-renewing axial progenitors that continue to generate neural and mesoderm tissues from the tail bud. This study identifies *Nkx1-2*-expressing cells as the source of most trunk and tail tissues in the mouse and provides a useful tool to genetically label and manipulate axial progenitors *in vivo*.

## Abstract (precis)

**Summary:** Changing lineage contributions of axial progenitors to the developing mouse embryo are revealed using a tamoxifen-inducible Cre line under the control of the endogenous *Nkx1-2* promoter.

## INTRODUCTION

The vertebrate body forms progressively in a head-to-tail direction from progenitors located at the posterior end of the embryo (reviewed by Kimelman, 2016; Neijts et al., 2013; Wilson et al., 2009). This axis elongation process is thought to be fuelled at least in part by a small pool of bipotential progenitors with self-renewing ability, the so-called neuromesodermal progenitors (NMPs) (Cambray and Wilson, 2002, 2007; Tsakiridis and Wilson, 2015; Tzouanacou et al., 2009). NMPs give rise to neural and mesodermal progenitors that form the spinal cord and paraxial mesoderm derivatives (e.g. bones, cartilage, muscle, dermis) of the trunk and tail (reviewed by Henrique et al., 2015; Steventon and Martinez Arias, 2017). Fate-mapping studies of small groups of cells in mouse embryos at embryonic day (E) 8.5 have shown that both the neural and paraxial mesoderm tissues of the trunk originate from the epiblast between the node and the anterior primitive streak (the node-streak border or NSB) and the caudal lateral epiblast (CLE) (Cambray and Wilson, 2007; Wymeersch et al., 2016). The cell population that retains the potential to generate new neural and mesodermal tissue is then contained within the region of the tail bud where the neural tube overlies the posterior end of the notochord – the chordo-neural hinge (CNH) (Cambray and Wilson, 2002; McGrew et al., 2008; Wilson and Beddington, 1996). These findings suggest that NMPs first reside within the NSB and CLE and after the trunk-to-tail transition are retained in the CNH.

Molecularly, NMPs have been defined by co-expression of the stem cell and neural transcription factor *Sox2* and the mesodermal transcription factor *T* (brachyury) (Garriock et al., 2015; Olivera-Martinez et al., 2012; Tsakiridis et al., 2014; Turner et al., 2014; Wymeersch et al., 2016). However, even though cells that co-express *Sox2* and *T* overlap with neuromesodermal-fated regions, co-expression of *Sox2* and *T* is not a feature unique to NMPs (Wymeersch et al., 2016). Recently, two studies revealed a more complete molecular signature of NMPs and their immediate descendants, early neural and mesodermal progenitors, using single-cell RNA-sequencing technologies (Gouti et al., 2017; Koch et al., 2017). Perhaps not surprisingly, both data sets showed that the CLE cell population (Gouti et al., 2017) and cells co-expressing *Sox2* and *T* at E8.5 (Koch et al., 2017) are rather heterogeneous and include, based on their molecular features, NMPs and early neural and mesodermal progenitors. NMPs at E8.5 express *Sox2*, *T*, *Nkx1-2*, *Cdx2* and *Cdx4*, and NMPs at E9.5 and NMPs undergoing lineage choice express NMP marker genes plus *Tbx6* at levels that reflect their fate choice (Gouti et al., 2017; Koch et al., 2017). Accordingly, early mesoderm progenitors express *T* and *Tbx6* and at decreasing levels *Sox2* and *Nkx1-2*, whereas early neural progenitors express *Sox2* and at decreasing levels *Nkx1-2* and *T*. Already committed presomitic mesoderm cells express *Msgn1* and *Tbx6* but have repressed *Sox2* and *Nkx1-2*, whereas neural progenitors express high *Sox2* but have now repressed *Nkx1-2* and mesodermal genes (Gouti et al., 2017; Koch et al., 2017). From these data, it emerges that *Nkx1-2* marks progenitor cells with neural and mesodermal potential. *Nkx1-2* has also been used to identify *in vitro*-derived NMPs (Edri et al., 2018 preprint; Gouti et al., 2014; Sasai et al., 2014; Tsakiridis et al., 2014; Verrier et al., 2018). *Nkx1-2*, previously *Sax1* in the chick, is a member of the small NK-l class of homeobox genes. *Nkx1-2* is widely conserved across species and its expression pattern has been characterised in chick (Rangini et al., 1989; Spann et al., 1994), mouse (Schubert et al., 1995) and zebrafish (Bae et al., 2004). However, the identity of *Nkx1-2*-expressing cells and their contributions to the developing mouse embryo have not been specifically characterised.

Here, we present the first detailed description of the expression pattern of *Nkx1-2* in the mouse embryo and show that it largely overlaps with the posterior growth zone and regions thought to harbour NMPs and early neural and mesodermal progenitors. We describe the generation and characterisation of the Nkx1-2CreER^T2^ transgenic mouse line in which tamoxifen-inducible CreER^T2^ recombinase is driven under the control of the endogenous *Nkx1-2* promoter. We then demonstrate that this line can be used to manipulate gene expression specifically in cells expressing *Nkx1-2* in a temporally controlled manner. Using a YFP reporter, we trace and define the lineages of the *Nkx1-2*-expressing cell population at different developmental stages and find that this progenitor population is dynamic, changing as development proceeds to supply most tissues of the trunk and tail in the mouse.

## RESULTS

### *Nkx1-2* is expressed in the posterior growth zone throughout body axis elongation

**Fig. 1.**
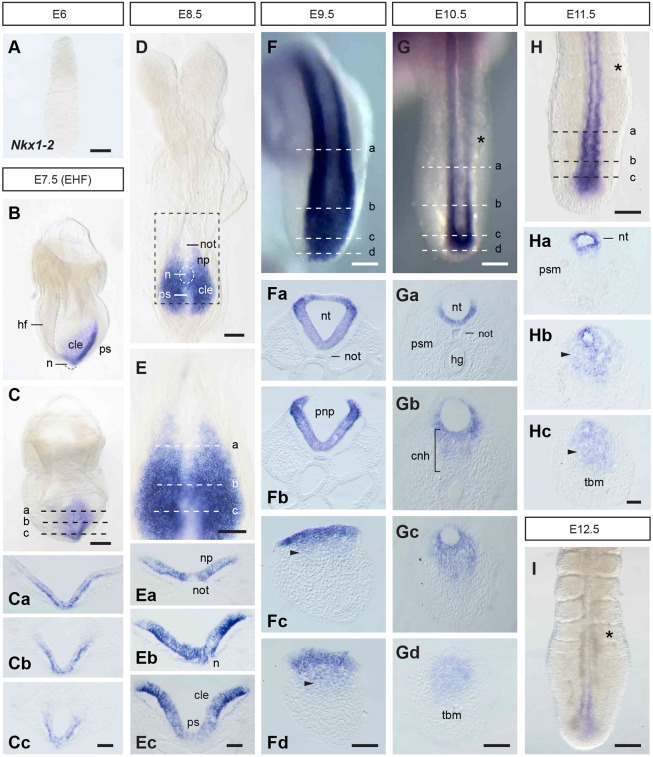
**Expression of *Nkx1-2* in the developing mouse embryo.** (A-C) *Nkx1-2* RNA *in situ* hybridisation of an E6.0 embryo (*n*=4) (A) and lateral (B) and posterior (C) views of an E7.5 embryo (early head fold, EHF) (*n*=4). (Ca-Cc) Transverse sections through the regions indicated in C. (D,E) *Nkx1-2* expression in an E8.5 embryo (D; 8-10 somites) and higher magnification of the posterior end of the embryo (boxed area; E) (*n*=4). (Ea-Ec) Transverse sections through the regions indicated in E. (F) Dorsal view of the posterior end of an E9.5 embryo (*n*=4). (Fa-Fd) Transverse sections through the regions indicated in F. (G) Dorsal view of the tail end of an E10.5 embryo (*n*=9). (Ga-Gd) Transverse sections through the regions indicated in G. (H) Dorsal view of the tail end of an E11.5 embryo (*n*=4). (Ha-Hc) Transverse sections through the regions indicated in H. (I) Dorsal view of the tail end of an E12.5 embryo (*n*=4). Arrowheads in Fc, Fd, Hb and Hc indicate the mesenchymal cell group expressing *Nkx1-2* in the tail bud. Asterisks in G, H and I indicate the last-formed somite. cle, caudal lateral epiblast; cnh, chordoneural hinge; hf, headfolds; hg, hindgut; n, node; not, notochord; np, neural plate; nt, neural tube; pnp, posterior neuropore; ps, primitive streak; psm, presomitic mesoderm; tbm, tail bud mesenchyme. Scale bars: 100 µm (whole-mount embryos); 50 µm (transverse sections).

To document in detail *Nkx1-2* expression in the mouse embryo, we carried out whole-mount RNA *in situ* hybridisation and then localised *Nkx1-2* transcripts to specific cell populations in serial transverse sections. As the body develops in a head-to-tail sequence, sections from the posterior end of the embryo represent less differentiated structures than more anterior sections. In agreement with a previous report (Schubert et al., 1995), *Nkx1-2* transcripts were first detected around E7-7.5 in the NSB as well as in and alongside the primitive streak, in cells of the CLE (Fig. 1A-C). This coincides with the emergence of the node and the time and regions in which NMPs first arise during embryonic development (Wymeersch et al., 2016). At E8.5, *Nkx1-2* expression remained highest in epiblast cells in the node region and CLE just posterior to the node (Fig. 1D,E,Eb,Ec). *Nkx1-2* was expressed at lower levels in the primitive streak, in cells that ingress to form mesoderm (Fig. 1Ec). Anterior to the node, *Nkx1-2* was also expressed in recently generated neural tissue, although at lower levels in the midline/floor plate (Fig. 1D,E,Ea). The expression pattern and relative levels of *Nkx1-2* in the E8.5 embryo combined with lineage-tracing data (Cambray and Wilson, 2007; Wymeersch et al., 2016) support single-cell transcriptomics data suggesting that *Nkx1-2* is highly expressed in NMPs and expressed at lower levels in early neural and mesodermal progenitors (Gouti et al., 2017; Koch et al., 2017). By E9.5, the most anterior *Nkx1-2*-expressing cells have begun to form a neural tube (Fig. 1F-Fb). Posteriorly, transcripts remained in epiblast cells around the closing posterior neuropore but were for the first time detected at lower levels in mesenchymal cells ingressing through the last remnants of the primitive streak as the tail bud forms (Fig. 1Fc,Fd). In the tail of E10.5 embryos, *Nkx1-2* transcripts continued to be detected in most newly formed neural tube (Fig. 1G-Gc) and were also found in the CNH region (Fig. 1Gb). Here, *Nkx1-2* was expressed in the neural tube and in a mesenchymal cell group continuous with the ventral neural tube, but not in the notochord component of the CNH (Fig. 1Gb). Posteriorly, *Nkx1-2* was also expressed in the contiguous dorsal tail bud mesenchyme, albeit at lower levels (Fig. 1Gd). Intriguingly, the appearance of this novel mesenchymal *Nkx1-2* domain coincides with the transition from primitive streak to tail bud-driven growth and formation of neural tissue by secondary neurulation, which involves a mesenchymal-to-epithelial transition (Beck, 2015; Lowery and Sive, 2004; Schoenwolf, 1984). At E11.5, *Nkx1-2* transcripts were still detected in the newly formed neural tube and contiguous tail bud mesenchyme (Fig. 1H). At all stages, the anterior limit of *Nkx1-2* expression was in the neural tube around the level of the last formed somite (Fig. 1D-H). At E12.5, when tail elongation is coming to a halt, *Nkx1-2* expression faded away (Fig. 1I). Outside of the posterior end of the embryo, *Nkx1-2* transcripts appeared at this stage in a subpopulation of motor neurons in the hindbrain and spinal cord and in the medial longitudinal fascicle of the midbrain (Schubert et al., 1995) (Fig. S1).

Taken together, these data show that *Nkx1-2* expression marks the posterior growth zone and regions thought to harbour NMPs and early neural and mesodermal progenitors throughout body axis elongation.

### *Nkx1-2* regions colocalise with SOX2^+^ T^+^ regions fated for neural and mesodermal lineages

NMPs are usually identified *in vivo* by their location and co-expression of the neural transcription factor SOX2 and the mesodermal transcription factor T (Garriock et al., 2015; Tsakiridis et al., 2014; Wymeersch et al., 2016). The relative levels of these two factors correlate with the fate of NMP descendants: neural-fated NMPs gradually increase *Sox2* and decrease *T* expression, whereas mesoderm-fated NMPs increase *T* and decrease *Sox2* (Gouti et al., 2017; Koch et al., 2017; Wymeersch et al., 2016). To better place NMP cells and their immediate descendants within *Nkx1-2* regions, we carried out SOX2 and T immunofluorescence on transverse sections of mouse embryos. Because embryos display a highly characteristic spatial patterning of tissues along the developing body axis, we used morphological features to align sections with schematics of the *Nkx1-2* regions defined above (Fig. 1). We focused the analysis around the regions known to harbour early and late NMPs – the NSB and CLE at E8.5 and the CNH at E10.5, respectively.

**Fig. 2.**
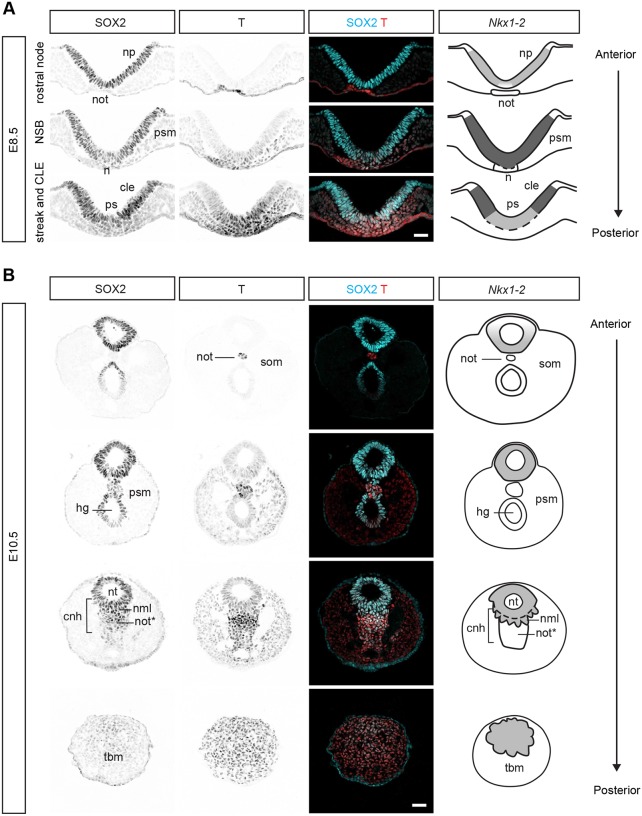
**SOX2 and T co-expression within *Nkx1-2* regions.** (A) Transverse sections across the rostral node, NSB and CLE of an E8.5 embryo immunolabelled for SOX2 and T (*n*=4). (B) Transverse sections across the tail end of an E10.5 embryo immunolabelled for SOX2 and T (*n*=7). The cartoons in (A) and (B) depict the expression pattern of *Nkx1-2* (as shown in Fig. 1). The different levels of *Nkx1-2* expression (based on *in situ* hybridisation signal) are represented by different grey intensities (light grey, low; dark grey, high). The dashed lines delineate regions not limited by basement membrane. Abbreviations are as in Fig. 1. nml, neuromesodermal lip; not*, notochord end; som, somite. Scale bars: 50 µm.

In agreement with previous reports (Garriock et al., 2015; Tsakiridis et al., 2014; Wymeersch et al., 2016), SOX2^+^ T^+^ cells were found at E8.5 at the midline epiblast of the NSB, and posteriorly in the CLE and primitive streak (Fig. 2A). Here, T levels were higher in the midline epiblast and primitive streak than in the CLE (Fig. 2A) (Wymeersch et al., 2016). Moreover, as recently reported (Javali et al., 2017), TBX6 could be detected in high-T regions – the primitive streak and primitive streak epiblast as well as in the presomitic mesoderm – but not in the neural plate or NSB epiblast, and only in few scattered cells in the CLE (Fig. S2). *Nkx1-2*, however, was expressed across these regions albeit at higher levels in the NSB epiblast and CLE than in the primitive streak epiblast (Fig. 1Ec, Fig. 2A). Taken together, these molecular features suggest that the *Nkx1-2*-expressing cell population at E8.5 includes putative NMPs (SOX2^+^ T^+^ TBX6^−^ *Nkx1-2*^high^) and early neural (SOX2^high^ T^−^ TBX6^−^ *Nkx1-2*^low^) and mesodermal (SOX2^low^ T^high^ TBX6^+^ *Nkx1-2*^low^) progenitors.

Between E9.5 and E10.5, NMPs become incorporated into the forming tail bud (Cambray and Wilson, 2002, 2007; Wilson and Beddington, 1996), but their precise position remains unclear. SOX2 and T co-expression is not unique for NMPs in the CNH region: node-derived notochord progenitors and hindgut cells also co-express SOX2 and T (Wymeersch et al., 2016), but they are not NMPs (Fig. 2B). However, by combining SOX2 and T with *Nkx1-2* expression data in the tissue context we could identify putative NMPs in the dorsal half of the CNH. These included cells located in the neural tube and mesenchymal cells directly below the neural tube (Fig. 2B). Given the co-expression of neural and mesodermal genes, we propose to name this medial mesenchymal cell population the neuromesodermal lip (Fig. 2B). In contrast, SOX2^+^ T^+^ cells in the ventral half of the CNH express T at higher levels and no or undetectable *Nkx1-2* and thus are mostly notochord progenitors (Fig. 2B). In agreement with a recent report (Javali et al., 2017), we found low but detectable levels of TBX6 protein in all SOX2^+^ cells in the CNH region, including *Nkx1-2*-expressing cells in the neural tube (higher in the ventral half) and in the neuromesodermal lip (Fig. S2). Posterior to the CNH, cells of the tail bud mesenchyme also co-express SOX2 and T proteins and low levels of *Nkx1-2* transcripts, resembling cells in the primitive streak epiblast at E8.5 (Fig. 2). Lineage tracing of dorsal tail bud mesenchyme (Cambray and Wilson, 2002; McGrew et al., 2008) and the molecular signature of this cell population, including TBX6 (Fig. S2), suggest that the *Nkx1-2*-expressing cells in the tail bud mesenchyme (*Nkx1-2*^low^ SOX2^low^ T^high^ TBX6^+^) are early mesoderm progenitors. As expected, presomitic mesoderm cells express high levels of T and TBX6 and neither SOX2 nor *Nkx1-2* (Fig. 2B, Fig. S2) (Chalamalasetty et al., 2014; Gouti et al., 2017).

Taken together, these data provide a refined map of the *in vivo* location of NMPs and their immediate descendants and identify *Nkx1-2* as a reliable marker for these dynamic progenitor cell populations throughout body axis elongation.

### Generation of Nkx1-2CreER^T2^/YFP reporter mice

**Fig. 3.**
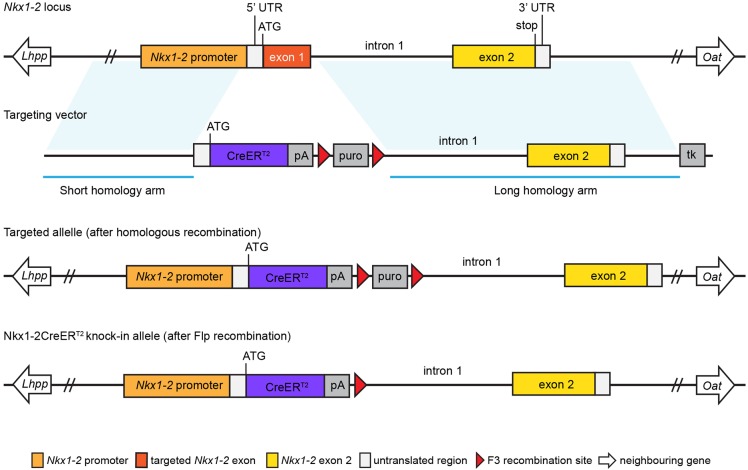
**Strategy to knock-in CreER^T2^ into the *Nkx1-2* locus.** *Nkx1-2* locus and targeting vector designed to replace *Nkx1-2* exon 1 and the splice donor site at the junction between exon 1 and intron 1 with a cassette containing the open reading frame of CreER^T2^. Recombinant clones were injected into mouse blastocysts and transferred to mice. The resulting chimeric mice were bred to Flp deleter mice, which ubiquitously express Flp recombinase, to remove the puromycin selection marker to generate the Nkx1-2CreER^T2^ line. The detailed map of the targeting vector and vector sequence can be found in Fig. S5 and Table S1. pA, polyadenylation site; puro, puromycin; tk, thymidine kinase.

To label and manipulate specifically *Nkx1-2*-expressing cells and thus potentially NMPs and early neural and mesodermal progenitors in a temporally controlled manner, we set out to generate a transgenic mouse line in which expression of CreER^T2^ recombinase is driven under the control of the *Nkx1-2* promoter. The strategy used to generate the conditional transgenic mouse is summarised in Fig. 3 (see also Materials and Methods).

To label fluorescently *Nkx1-2*-expressing cells in developing embryos, homozygous Nkx1-2CreER^T2^ females were crossed with heterozygous or homozygous males harbouring a *loxP*-flanked stop sequence upstream of the EYFP reporter gene under the control of the ubiquitous *ROSA26* promoter (R26R*-*EYFP mice) (Srinivas et al., 2001). In the resulting Nkx1-2CreER^T2^ floxed EYFP mice (Nkx1-2CreER^T2^/YFP), tamoxifen administration leads to CreER^T2^-mediated recombination of the *loxP*-flanked stop sequence and expression of the YFP reporter in *Nkx1-2*-expressing cells and their progeny. We confirmed that Nkx1-2CreER^T2^ faithfully drives transgene expression in the endogenous *Nkx1-2* regions (Figs S3 and S4A-B) and that in the absence of tamoxifen only low levels of spontaneous recombination occur, and always within canonical *Nkx1-2* regions (Fig. S4C). Thus, overall, tamoxifen-induced CreER^T2^-mediated recombination leads to faithful YFP-labelling of *Nkx1-2*-expressing cells in Nkx1-2CreER^T2^/YFP reporter mice.

### Nkx1-2CreER^T2^/YFP labels SOX2 and T co-expressing cells and their progeny

**Fig. 4.**
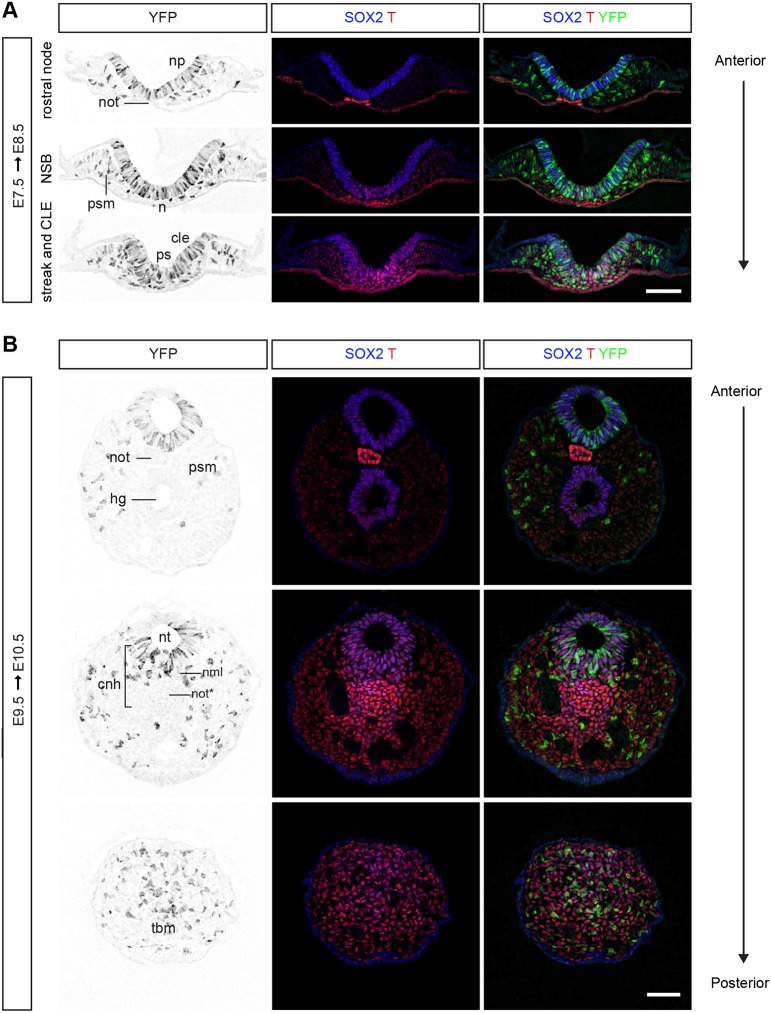
**A subset of *Nkx1-2*-expressing cells and/or their progeny express SOX2 and T.** (A) Transverse sections through the rostral node, NSB and CLE of an E8.5 Nkx1-2CreER^T2^ embryo that was exposed to tamoxifen at E7.5 and immunolabelled for SOX2, T and YFP (*n*=7). (B) Transverse sections through the tail end of an E10.5 Nkx1-2CreER^T2^ embryo that was exposed to tamoxifen at E9.5 and immunolabelled for SOX2, T and YFP (*n*=9). Abbreviations are as in Fig. 1. nml, neuromesodermal lip; not*, notochord end. Scale bars: 100 µm.

**Fig. 5.**
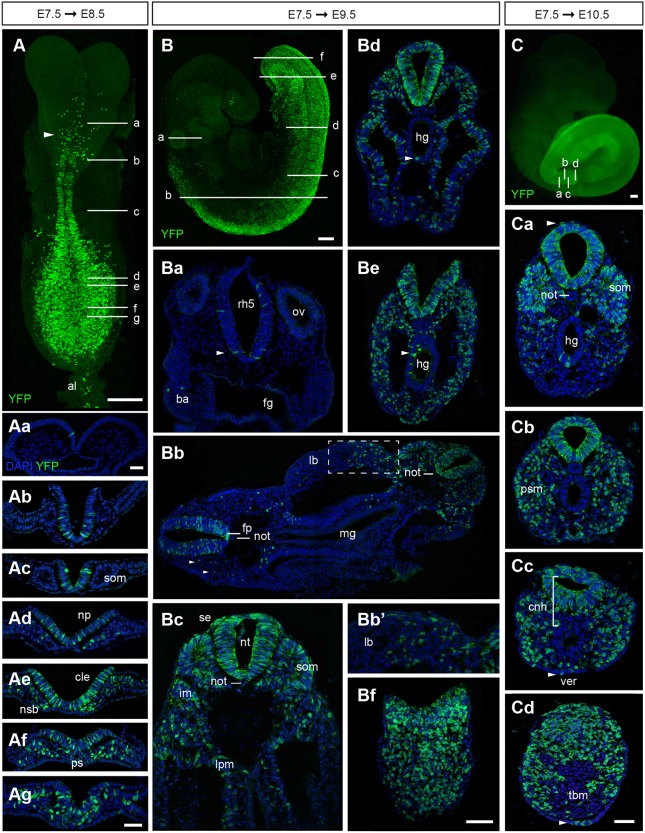
**Lineage tracing of cells expressing *Nkx1-2* at E7.5.** Timed-pregnant Nkx1-2CreER^T2^ mice received tamoxifen at E7.5 and the contribution of YFP^+^ cells to developing embryos was assessed at E8.5, E9.5 and E10.5. (A) Maximum intensity projection (MIP) of an E8.5 embryo immunolabelled for YFP on whole-mount (*n*=7). The arrowhead marks the presumptive midbrain/anterior hindbrain boundary. (Aa-Ag) Transverse sections through the regions indicated in A (*n*=8). (B) Composite MIP of a E9.5 embryo immunolabelled for YFP on whole-mount (*n*=16, 14 of 16 embryos analysed had YFP^+^ cells in the eye). (Ba-Bf) Transverse sections through the regions indicated in B (*n*=8). (Ba) At the level of the otic vesicles (ov) and rhombomere 5 (rh5) scattered YFP^+^ cells were found in the neural tube (arrowhead). A few YFP^+^ cells populated the branchial arches (ba). The foregut (fg) was, however, always unlabelled. (Bb) Section comprising the anterior (left) and posterior (right) levels of the trunk. YFP^+^ cells were found scattered across the more anterior neural tube, including the floor plate (fp), and spanned the dorsoventral extent of the posterior neural tube. YFP^+^ cells generated neural crest cells (arrowheads), contributed to posterior somites, and to limb bud (lb) mesenchyme. YFP^+^ cells were absent from the notochord (not) and midgut (mg). (Bb′) Higher magnification of limb bud mesenchyme in Bb (dashed box). (Bc) YFP^+^ cells contributed to the neural tube (nt), somites (som), intermediate mesoderm (im), lateral plate mesoderm (lpm) and surface ectoderm (se). (Bd, Be) YFP^+^ cells were found frequently in the hindgut (hg) (arrowheads). (Bf) YFP^+^ cells extended to the caudal epiblast and underlying mesenchyme. (C) Wide-field fluorescence image of an E10.5 embryo that received tamoxifen at E7.5. Most of the posterior body derived from YFP^+^ cells at this stage (*n*=7). (Ca) YFP^+^ cells made most of the neural tube and somites (som) and also contributed to hindgut (hg) endoderm and surface ectoderm (arrowhead), but were absent from the notochord (not). (Cb) Posterior to Ca, most presomitic mesoderm (psm) is YFP^+^. (Cc) In addition to the neural tube and paraxial mesoderm, YFP^+^ cells were found in the neuromesodermal lip in the CNH (cnh) region. YFP^+^ cells were also present in the VER (ver, arrowhead). (Cd) The tail bud mesenchyme (tbm) except the ventral-medial cell group was YFP^+^. The images are representative images of each stage and anterioposterior level. al, allantois; cle, caudal lateral epiblast; np, neural plate; nsb, node-streak border; ps, primitive streak. Scale bars: 100 µm (whole-mount embryos); 50 µm (transverse sections).

To establish which cells are labelled with the Nkx1-2CreER^T2^/YFP reporter and whether they include NMPs, we set out to identify YFP^+^ cells based on their location and expression of SOX2 and T. Timed-pregnant Nkx1-2CreER^T2^/YFP mice received tamoxifen either at E7.5 (the onset of *Nkx1-2* expression) or E9.5 (when axial progenitors transition into the forming tail bud) to label *Nkx1-2*-expressing cells around these stages; 24 h later we analysed the posterior growth zone in the epiblast and tail bud. In embryos exposed to tamoxifen at E7.5 and analysed at E8.5, most SOX2^+^ T^+^ cells in the epiblast layer of the NSB and CLE were also YFP^+^. This suggests that Nkx1-2CreER^T2^/YFP labels putative NMPs (Fig. 4A). As would then be expected, YFP^+^ cells were also found in the neural plate (SOX2^+^ T^−^), ingressing mesoderm, and paraxial mesoderm (SOX2^−^ T^+^) (Fig. 4A). Additionally, a few YFP^+^ cells were found in intermediate and lateral plate mesoderm as well as prospective surface ectoderm (Fig. 4A). These findings indicate that the *Nkx1-2*-expressing cell population in the epiblast around E7.5 is heterogeneous, composed of NMPs and early neural and paraxial mesoderm progenitors, lateral plate and intermediate mesoderm progenitors, as well as a few endoderm-fated progenitors. In embryos exposed to tamoxifen at E9.5 and analysed at E10.5, a subset of YFP^+^ cells co-expressed SOX2 and T in the dorsal half of the CNH, including the neural tube and the neuromesodermal lip (Fig. 4B). YFP^+^ cells were, however, absent from the notochord component of the CNH (SOX2^+^ T^high^ cells) and the hindgut (also SOX2^+^T^+^). In addition to the dorsal half of the CNH, YFP^+^ cells also populated the contiguous tail bud mesenchyme (Fig. 4B) and contributed to NMP lineages: most newly formed neural tube and paraxial mesoderm (Fig. 5). Overall, using the Nkx1-2CreER^T2^/YFP reporter it is possible to label specifically axial progenitors, including NMPs and their most immediate descendants, at defined developmental stages. These short-term tracing experiments also suggested that the *Nkx1-2*-expressing cell population is dynamic, changing its lineage contributions as development proceeds.

### Early *Nkx1-2*-expressing cells contribute to all three germ layers

To investigate the long-term contribution of early *Nkx1-2-*expressing cells and their progeny to the developing mouse embryo, timed-pregnant Nkx1-2CreER^T2^/YFP mice received a single dose of tamoxifen at E7.5 and the contribution of YFP^+^ cells was assessed in embryos at progressively later developmental stages. Assessment of embryos at E8.5 revealed scattered single cells across the presumptive midbrain/anterior hindbrain as the anterior limit of cells derived from *Nkx1-2*-expressing cells (Fig. 5A,Aa). YFP^+^ cells were found contiguously in the neural tube from the presumptive posterior hindbrain (Fig. 5A,Ab) and then, more posteriorly, throughout the CLE and primitive streak (Fig. 5A,Ad-Ag). YFP^+^ cells were also present in derivatives of the primitive streak – the recently ingressed mesoderm and most-recently formed presomitic mesoderm – but were absent from the first four or five somites (Fig. 5Ad-Ag). At the posterior end of the embryo, a few YFP^+^ cells also contributed to intermediate mesoderm and lateral plate mesoderm compartments as well as the allantois (Fig. 5A,Af,Ag). YFP^+^ cells were consistently absent from the notochord (Fig. 5A,Ac).

A day later, in E9.5 embryos, again a few scattered cells were located in the midbrain and the roof of the anterior hindbrain as well as in the developing eye (Fig. 5B). The anterior limit of contiguous YFP labelling was now clearly located in the hindbrain just anterior to the otic vesicle in rhombomere 5 (Fig. 5B). More posteriorly, YFP^+^ cells were concentrated ventrally, including the floor plate of the spinal cord (Fig. 5Ba,Bc). This finding suggests that the floor plate of the trunk spinal cord originates from cells expressing *Nkx1-2*, probably from the dorsal layer of the node (see Fig. 1Eb), as indirectly suggested by the combined results of earlier cell labelling studies in mice (Beddington, 1994; Sulik et al., 1994) and in the chick (Selleck and Stern, 1991). From forelimb levels to the posterior end of the embryo, YFP^+^ cells were found throughout the dorsoventral extent of the neural tube, in somites and their derivatives (Fig. 5Bb,Bc). YFP^+^ cells also contributed extensively to intermediate and lateral plate mesoderm (Fig. 5B,Bc). Mesenchymal cells derived from the lateral plate mesoderm could be seen migrating into the limb bud (Fig. 5Bc). From forelimb levels, YFP^+^ cells also appeared in the surface ectoderm (Fig. 5Bc-Bf) and as streams of neural crest cells emerging from the dorsal neural tube (Fig. 5Bc). YFP^+^ cells were absent from the first four or five somites, but contributed to both medial and lateral compartments of the posterior-most 11-12 somites (Fig. 5B,Bb,Bd). YFP^+^ cells did not contribute to the notochord (Fig. 5Ba-Bc), although a few isolated YFP^+^ cells were found in the notochord of one embryo. This finding argues against a common source of floor plate and notochord after E7.5 and agrees with grafting and cell labelling experiments that indicated that the ventral node, which does not express *Nkx1-2* (see Fig. 1Eb), is the source of trunk notochord (Beddington, 1994; Brennan et al., 2002; Yamanaka et al., 2007). In all E9.5 embryos examined, YFP^+^ cells were absent from the fore- and midgut (Fig. 5Ba-Bc), but frequently found in the hindgut (Fig. 5Bd,Be). At the posterior end of the embryo, YFP^+^ cells constituted most of the posterior neuropore and underlying mesenchyme (Fig. 5Bc-Bf). Overall, these lineage-tracing studies show that the majority of descendants of E7.5 *Nkx1-2*-expressing cells contribute to the neural and mesodermal tissues of the trunk, including paraxial, intermediate and lateral plate mesoderm as well as to the extra-embryonic allantois.

Between E9.5 and E10.5, axial progenitors complete trunk formation and begin forming a tail. In E10.5 Nkx1-2CreER^T2^/YFP embryos exposed to tamoxifen at E7.5, YFP^+^ cells made up most of the neural tube and paraxial mesoderm/somites of the tail (Fig. 5C-Cd). Posteriorly, YFP^+^ cells populated the dorsal half of the CNH (Fig. 5Cc) and the presomitic mesoderm as well as the tail bud mesenchyme contiguous with these regions (Fig. 5Cd). YFP^+^ cells were, however, virtually absent in the ventral compartment of the CNH and the ventral tail bud mesenchyme (Fig. 5Cd). YFP^+^ cells were found very rarely in the tail notochord and occasionally in the hindgut (Fig. 5C-Cc). A few YFP^+^ cells contributed to surface ectoderm and to the ventral ectodermal ridge (VER) (Fig. 5Cc). Importantly, as tamoxifen persists for less than 24 h in our system (see Materials and Methods), this long-term lineage-tracing experiment indicates that cells that expressed *Nkx1-2* at E7.5 and/or their progeny persist in the later posterior growth zone. This supports the possibility that at least some *Nkx1-2*-expressing cells self-renew in this region from where they then continue to generate neural and mesoderm tissues.

### Late *Nkx1-2*-expressing cells continue to make neural and mesodermal tissues from the tail bud

**Fig. 6.**
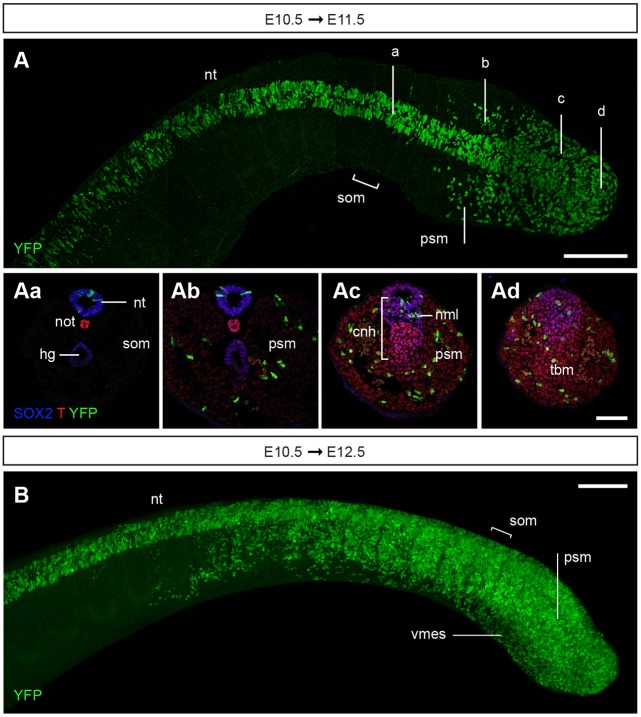
**Lineage tracing of cells expressing *Nkx1-2* at E10.5.** (A,B) Timed-pregnant Nkx1-2CreER^T2^ mice received tamoxifen at E10.5 and the contribution of YFP^+^ cells to developing embryos was assessed at E11.5 (A) and E12.5 (B). (A) Dorsal view (composite MIP) of the tail of an E11.5 embryo immunolabelled for YFP on whole-mount (*n*=7). (Aa-Ad) Transverse sections representative of the levels indicated in A and immunolabelled for SOX2, T and YFP (*n*=9). YFP^+^ cells contributed to the secondary neural tube (Aa-Ad), presomitic mesoderm (Ab-Ad) and tail bud mesenchyme (Ad). (B) Side view (MIP) of the tail of an E12.5 embryo immunolabelled for YFP on whole-mount (*n*=9). Abbreviations are as in Fig. 1. vmes, ventral tail bud mesoderm. Scale bars: 100 µm (whole-mount embryos); 50 µm (transverse sections).

To test whether the *Nkx1-2*-expressing cell population in the tail bud retains the ability to contribute neural and mesodermal lineages, timed-pregnant Nkx1-2CreER^T2^/YFP mice received tamoxifen at E10.5 and embryos were assessed 24 or 48 h later. In E11.5 embryos, YFP^+^ cells were indeed found in the neural tube and paraxial mesoderm as well as in the tail bud mesenchyme (Fig. 6A). YFP^+^ cells in the neural tube extended from the tail bud to axial levels right below the hindlimb (opposite to somite ∼36), where the transition from trunk to tail development and from primary to secondary neurulation takes place (Shum et al., 2010). YFP^+^ cells in the paraxial mesoderm were found more posteriorly, in newly generated presomitic mesoderm (Fig. 6A). This different distribution of YFP^+^ cells in the neural tube and paraxial mesoderm likely reflects the broader expression of *Nkx1-2* in the newly formed neural tube (Fig. 1G). *Nkx1-2*-expressing cells labelled at E9.5 did not contribute to surface ectoderm or the hindgut (Fig. 4B) and, perhaps not surprisingly, cells labelled at E10.5 did not contribute to these tissues in E11.5 embryos either (Fig. 6A). Analysis of embryos at E12.5, 48 h after tamoxifen administration, confirmed that cells expressing *Nkx1-2* at E10.5 contribute to both the neural tube and paraxial mesoderm/somites of the tail, while also being retained in the tail bud (Fig. 6B). These findings indicate that the *Nkx1-2*-expressing cell population in the tail bud continues to generate neural and mesoderm tissues and are consistent with the possibility that some of these cells self-renew until the end of body axis elongation.

## DISCUSSION

Axial progenitors, including NMPs, have proved challenging to study in the developing mouse embryo because they comprise a relatively small (Wymeersch et al., 2016), dynamic (this study; Aires et al., 2016; Gouti et al., 2017; Javali et al., 2017; Koch et al., 2017) and transient cell population, which lacks unique molecular markers. Lineage-tracing studies involving dye labelling or the grafting of small groups of cells can be imprecise because of the close proximity between various types of progenitors and extensive cell mixing in the rapidly growing embryo. A complementary approach is to use a well-documented Cre reporter mouse that can be used to target just axial progenitors *in vivo*. In this study, we elucidate the expression pattern of *Nkx1-2* in the mouse embryo and show that it is transcribed in axial progenitors throughout body axis elongation. Using *Nkx1-2* along with other markers, we then map the molecular heterogeneity of axial progenitor cell populations in the embryo. By creating a tamoxifen-inducible Nkx1-2CreER^T2^ transgenic mouse, we label and trace *Nkx1-2*-expressing cells at specific stages during development. We show that early *Nkx1-2*-expressing cells contribute to all three germ layers, mostly to neural and mesodermal lineages (excluding notochord), but also to surface ectoderm and hindgut endoderm, whereas late *Nkx1-2-*expressing cells generate the neural and paraxial mesoderm tissues of the tail. Importantly, the presence of YFP^+^ cells in the tail bud after much earlier tamoxifen exposure, is consistent with the notion that some axial progenitors have self-renewing ability.

### *Nkx1-2* identifies axial progenitors, including NMPs and early neural and mesodermal progenitors

Our analysis of *Nkx1-2* expression during embryonic development identified this gene as an enduring marker of the posterior growth zone. Using a set of defining molecular markers, we further characterised the molecular heterogeneity within *Nkx1-2*-expressing regions and demonstrated that these encompass early neural and mesodermal progenitors as well as NMPs. The expression of *Nkx1-2* is highest in SOX2^+^ T^+^ cells in the epiblast, which are commonly defined as NMPs. Heterochronic grafting experiments suggest that NMPs later reside within the CNH region of the tail bud (Cambray and Wilson, 2002); however, SOX2 and T co-expression is not unique to the CNH in the tail bud. Our definition of the *Nkx1-2* expression domain within the CNH helped to distinguish this SOX2^+^ T^+^ cell population. Indeed, *Nkx1-2* expression is initially localised within the CNH in an intriguing group of mesenchymal cells, which we have called the neuromesodermal lip. These cells have morphological and molecular resemblance to cells emerging from the earlier primitive streak. As recently reported and indeed similar to the late primitive streak, cells in the neuromesodermal lip additionally express TBX6 (Javali et al., 2017; this study). This molecular signature (SOX2^+^ T^+^ *Nkx1-2*^+^ TBX6^+^) is also similar to that of NMPs at E9.5 deduced from single-cell transcriptomics data (Gouti et al., 2017). Overall, our detailed analyses document the heterogeneity within the early *Nkx1-2*-expressing cell population and a distinct change in the molecular identity of the *Nkx1-2*-expressing cells that are internalised to form the tail bud.

It is noteworthy that *Nkx1-2* expression at the posterior end of the E8.5 embryo much resembles the region of transcriptional activity of the *Sox2* N1 enhancer (Takemoto et al., 2011). The expression of the *Sox2* N1 enhancer and *Nkx1-2* are both promoted by FGF signalling (Delfino-Machín et al., 2005; Sasai et al., 2014; Takemoto et al., 2011), which acts together with Wnt signalling to regulate and maintain the NMP pool in the embryo (Garriock et al., 2015; Jurberg et al., 2014; Wymeersch et al., 2016). This suggests that the expression of *Nkx1-2* identifies the broader axial progenitor cell state. Importantly, as discussed above, cells that co-express *Nkx1-2*, SOX2 and T in the tail bud now all express TBX6. This has recently been suggested to represent a transition state in NMPs undergoing lineage choice (Gouti et al., 2017; Javali et al., 2017; Koch et al., 2017) and interestingly coincides with the loss of the pluripotency factor *Oct4* (*Pou5f1*) in progenitors as the tail bud forms (Aires et al., 2016). As TBX6 represses the activity of the *Sox2* N1 enhancer associated with the NMP state (Takemoto et al., 2011), it would be interesting to determine whether this regulatory element is repressed in the tail bud or if it remains active in the cells that express *Nkx1-2*. One possibility is that such changes on tail bud formation underpin the subsequent gradual diminution of the NMP pool and so axial progenitors, which ultimately leads to the end of axis elongation.

### *Nkx1-2*-expressing cells generate most of the tissues of the trunk and tail

Our long-term lineage tracing of cells expressing *Nkx1-2* at E7.5 revealed that this cell population includes progenitors for most trunk tissues: neuroectoderm and almost all mesoderm tissues (paraxial, intermediate and lateral plate mesoderm, except notochord) as well as some progenitors for surface ectoderm and hindgut. Interestingly, a recent study derived an NMP-like cell population from mouse epiblast stem cells and found that such cells possess the potential to differentiate not only into neural and paraxial mesoderm cells but also into intermediate and lateral plate mesoderm (Edri et al., 2018 preprint). Our findings confirm that such a cell population exists in the E7.5 embryo and that it is included in the *Nkx1-2-*expressing cell population. Importantly, *Nkx1-2-*expressing cells labelled after E8.5 no longer contribute to endoderm (Lawson et al., 1991; Lawson and Pedersen, 1992; Tam and Beddington, 1987; Wilson and Beddington, 1996). This change in lineage contribution is also apparent in our data, with only labelled cells found in the hindgut following tamoxifen exposure at E7.5. This experiment also revealed that E7.5 *Nkx1-2*-expressing cells contribute to surface ectoderm, whereas cells labelled at E9.5 no longer contribute to hindgut or surface ectoderm. Labelling of late *Nkx1-2*-expressing cells in the E10.5 tail bud reveals that this late progenitor pool continues to generate neural and paraxial mesoderm tissues, but no longer contains progenitors for intermediate or lateral plate mesoderm, which are used up during trunk development to generate the kidney and gonads and their respective duct systems, as well as the circulatory system (Aires et al., 2016; Jurberg et al., 2013). This gradual restriction in the lineage contributions of the *Nkx1-2*-expressing cell population during body axis elongation most likely reflects the changing environment that the progeny of axial progenitors encounters in the elongating embryo. Indeed, elegant grafting experiments have further demonstrated that the contribution of axial progenitors to neural or mesodermal fates is influenced by where they are located within the NSB and later within the tail bud (Cambray and Wilson, 2007; Wymeersch et al., 2016).

Experiments in which we labelled *Nkx1-2*-expressing cells at E7.5 and assessed embryos at progressively later stages demonstrated that that some axial progenitors and/or their progeny are retained in the posterior growth zone as the embryo elongates. Further, labelled cells were retained in the E12.5 tail bud following late exposure to tamoxifen at E10.5. These findings support the idea that some axial progenitors remain in the tail bud and undergo self-renewal, as proposed following retrospective clonal analysis carried out in the mouse embryo (Tzouanacou et al., 2009). The Nkx1-2CreER^T2^ transgenic mouse line provides the opportunity to dissect further how these progenitors form the posterior body in the developing mouse embryo. For example, crossing the Nkx1-2CreER^T2^ mouse line with a multicolour reporter such as the R26R-Confetti mouse (Snippert et al., 2010) will allow detailed analysis of the contributions of single axial progenitors. This would address the issue of whether individual cells retained in the posterior growth zone do indeed self-renew and contribute to both neural and mesodermal lineages throughout body axis elongation. Crossing the Nkx1-2CreER^T2^ mouse line, with conditional knock-in or knockout mice is already providing new mechanistic insights into how this important cell population is directed to form trunk and tail tissues at the right place and time (Mastromina et al., 2018; Nikolopoulou et al., 2017; Rolo et al., 2016).

## MATERIALS AND METHODS

All animal procedures were performed in accordance with UK and EU legislation and guidance on animal use in bioscience research. This work was carried out under the UK project license 60/4454 and was subjected to local ethical review.

### Mice

Wild-type CD-11 and C567BL/6J mouse strains and transgenic lines Nkx1-2CreER^T2^, R26R-EYFP (Srinivas et al., 2001) and Nkx1-2CreER^T2^/YFP were maintained on a 14-h light/10-h dark cycle. For timed matings, the morning of the plug was considered E0.5. Gastrula embryos were staged according to Downs and Davies (1993) and Lawson and Wilson (2016) and at later stages by standard morphological criteria.

### Generation of Nkx1-2CreER^T2^ transgenic mice

The *Nkx1-2* coding sequence in exon 1 and the splice donor site at the junction between exon 1 and intron 1 were replaced with the open reading frame of tamoxifen-inducible CreER^T2^ recombinase as depicted in Fig. 3. The genomic sequence downstream of the insertion site was left intact to preserve all potential regulatory elements driving expression of the *Nkx1-2* gene. To do so, a targeting vector was generated by Taconic Biosciences using BAC clones from the C57BL/6J RPCIB-731 BAC library. The targeting vector included two homology arms 3.5 kb upstream of the *Nkx1-2* exon 1 and 5.4 kb downstream of the junction between exon 1 and intron 1, a cassette containing the open reading frame of CreER^T2^ recombinase, a polyadenylation signal 3′ of the CreER^T2^ sequence, and two selection markers: puromycin, flanked by F3 sites, and thymidine kinase (see Fig. S5 for the map of the targeting vector and Table S1 for the sequence). The targeting vector was linearised using *Not*I and transfected into the TaconicArtemis C57BL/6N Tac embryonic stem cell line and homologous recombinant clones were isolated using positive (puromycin resistance) and negative (thymidine kinase) selection. Fourteen targeted clones carried the inserted CreER^T2^ cassette and four of them (B-E1, BH9, B-H11, C-F5) were further validated by Southern blot and PCR analysis (Figs S6 and S7, Tables S2 and S3). Homologous recombinant clones were transfected into mouse blastocysts and transferred to mice. Taconic provided two breeding pairs of heterozygous C57BL/6–Nkx1-2^tm2296(Cre-ER(T2))Arte^ mice. These mice carried a puromycin-expressing cassette flanked by F3 sites, which was removed upon crossing to Flp-expressing mice. The resulting mice express CreER^T2^ from the endogenous *Nkx1-2* promoter (Nkx1-2CreER^T2^). Nkx1-2CreER^T2^ mice were then bred to homozygosity to establish a breeding colony. Loss of function of the *Nkx1-2* gene did not generate a phenotype in either heterozygous or homozygous mice. This is likely due to genetic compensation or functional redundancy by another related gene/s (but not the paralogue gene *Nkx1-1*, see Data S1 and Fig. S8). We have now maintained the Nkx1-2CreER^T2^ colony for more than nine generations without any obvious deleterious effects.

### Genotyping

Genotyping by standard methods was performed to maintain the homozygous line using the following PCR conditions: 95°C, 5 min and then 95°C, 30 s; 60°C, 30 s; 72°C, 1 min for 35 cycles followed by 72°C, 10 min. A DNA quality control and a test reaction were carried out in parallel for the knock-in (KI) allele, the wild-type (WT) allele, and the Flpe deleter (TG) using the following primer pairs: KI primer 1, 5′-ACGTCCAGACACAGCATAGG-3′; KI primer 2, 5′-TCACTGAGCAGGTGTTCAGG-3′ (fragment size 279 bp); QC primer 3, 5′-GAGACTCTGGCTACTCATCC-3′; QC primer 4, 5′-CCTTCAGCAAGAGCTGGGGAC-3′ (fragment size 585 bp); WT primer 5, 5′-CAAGGTTTATTGGTAGCCTGG-3′; WT primer 6, 5′-TGAGCCAGTCAGAGTTGTGG-3′ (fragment size 176 bp); QC primer 7, 5′-GTGGCACGGAACTTCTAGTC-3′; QC primer 8, 5′-CTTGTCAAGTAGCAGGAAGA-3′ (fragment size 335 bp); TG primer 9, 5′-GGCAGAAGCACGCTTATCG-3′; TG primer 10, 5′-GACAAGCGTTAGTAGGCACAT-3′ (fragment size 343 bp); QC primer 3 as above, QC primer 4 as above (fragment size 585 bp).

### Tamoxifen administration

To make a tamoxifen stock solution, tamoxifen powder (Sigma-Aldrich, T5648) was dissolved in vegetable oil to a final concentration of 40 mg/ml and sonicated to bring to solution. The tamoxifen stock solution was stored at −20°C for up to 3 months. At various stages of pregnancy, Nkx1-2CreER^T2^/YFP females were given a single 200 µl dose of tamoxifen (of the 40 mg/ml stock) by oral gavage. Mice were monitored for 6 h and when required were killed following schedule 1 of the Animals (Scientific Procedures) Act of 1986. Following this protocol, tamoxifen-induced recombination in Nkx1-2CreER^T2^/YFP mice occurred for less than 24 h because administration of tamoxifen at E6.5 (about 24 h before the onset of *Nkx1-2*/CreER^T2^ expression) did not result in numbers of YFP-labelled cells above those seen in embryos that did not receive tamoxifen at all (Fig. S4C).

### RNA *in situ* hybridisation

Embryos were dissected in ice-cold PBS and fixed in ice-cold 4% paraformaldehyde (PFA) overnight at 4°C. Standard methods were used to carry out mRNA *in situ* hybridisation in wild-type CD-1 and C57BL/6J (Charles River) mouse embryos (Wilkinson and Nieto, 1993). The *Nkx1-2* plasmid was kindly provided by Frank Schubert (Schubert et al., 1995). This probe includes the homeobox domain and the 3′ half of the gene (nucleotides 504-1057). The Cre plasmid was kindly provided by Anna-Katerina Hadjantonakis (Memorial Sloan Kettering Cancer Center, USA) (Kwon and Hadjantonakis, 2009).

### Immunofluorescence and imaging

Embryos were dissected in ice-cold PBS and fixed in ice-cold 4% PFA for 2 h. Embryos were then washed in PBS and permeabilised by dehydration in an increasing methanol series (25% methanol/PBS, 50% methanol/PBS, 75% methanol/PBS, 100% methanol), then stored in 100% methanol at −20°C, or bleached in 3% H_2_O_2_/methanol and gradually rehydrated in PBS in preparation for immunofluorescence. For whole-mount immunofluorescence, whole embryos were blocked in PBS/0.1% Triton X-100 (PBST) and 10% normal donkey serum (NDS) for 4 h and incubated with primary antibodies in PBST/NDS (1:500) overnight at 4°C. After incubation with primary antibodies, embryos were washed extensively in PBST (throughout the day or until the next day) and then incubated with secondary antibodies (1:500) and DAPI (1 mg/ml stock solution diluted 1:500) in PBST/10% NDS overnight at 4°C. Embryos were then washed extensively for 24 h and prepared for clearing. For BABB (2:1 benzyl alcohol:benzyl benzoate) clearing, embryos were first dehydrated in an increasing methanol series (25% methanol/PBS, 50% methanol/PBS, 75% methanol/PBS, 100% methanol, 5 min each), then put in 1:1 (v/v) methanol:BABB for 5 min and twice in BABB for clearing. BABB-cleared embryos were mounted in BABB for imaging. For immunofluorescence on cryosections, embryos were cryoprotected in 30% sucrose/PBS overnight at 4°C, mounted in agar blocks (1.5% agar/5% sucrose/PBS), and frozen on dry ice. Sections (16 µm thick) were cut on a Leica CM1900 cryostat, mounted on adhesion slides, and dried for several hours at room temperature. Slides were then washed three times in PBST and blocked in PBST/10% NDS at room temperature. After at least 1 h, sections were incubated with primary antibodies in PBST/10% NDS overnight at 4°C. After several PBST washes, sections were incubated with secondary antibodies and DAPI in PBST/10% NDS for 2 h at room temperature or overnight at 4°C. After several PBST washes, slides were mounted with SlowFade Gold antifade mountant (Invitrogen, S36936) for imaging.

Primary antibodies and working dilutions used were: chicken anti-GFP (Abcam, ab13970; 1:500), goat anti-GFP (Abcam, ab6673; 1:500), rabbit anti-SOX2 (Millipore, AB5603; 1:500), goat anti-SOX2 (Immune Systems, GT15098; 1:500), goat anti-brachyury/T (R&D Systems, AF2085; 1:500), goat anti-TBX6 (R&D Systems, AF4744; 1:200). Secondary antibodies used (all at 1:500) were: donkey anti-chicken Alexa Fluor 488 (Abcam, ab150173), donkey anti-goat Alexa Fluor 488 (Life Technologies, A11055), donkey anti-rabbit Alexa Fluor 568 (Life Technologies, A10042), donkey anti-goat Alexa Fluor 647 (Life Technologies, A21477).

Whole-mount embryos and tissue sections were imaged on a Leica TCS SP8 confocal laser scanning microscope in the Dundee Imaging Facility. Tissue sections were in some cases scored on a Leica DB fluorescence microscope or with a DeltaVision imaging system. Composite images were stitched together using the stitching algorithm in the Leica Application Suite X (LAS X) software and all images were prepared for publication using Fiji (fiji.sc/Fiji) (Schindelin et al., 2012).

### Methodology

The sample size of each experiment is reported in its respective figure legend. In all cases, *n* reflects the number of embryos analysed per experiment. All experiments were repeated at least twice (so embryos are from at least two independent litters). No statistical methods were used to predetermine sample size. The experiments were not randomised and the investigators were not blinded during the group allocation or outcome assessment.

## Supplementary Material

Supplementary information
