# Supplementary material for: Lineage tracing of axial progenitors using Nkx1-2CreERT2 mice defines their trunk and tail contributions
Source: Development. 2018 Oct 2;145(19):dev164319. doi: 10.1242/dev.164319 (PMC6198475; doi:10.1242/dev.164319)
Supplement: Supplementary information [file develop-145-164319-s1.pdf]

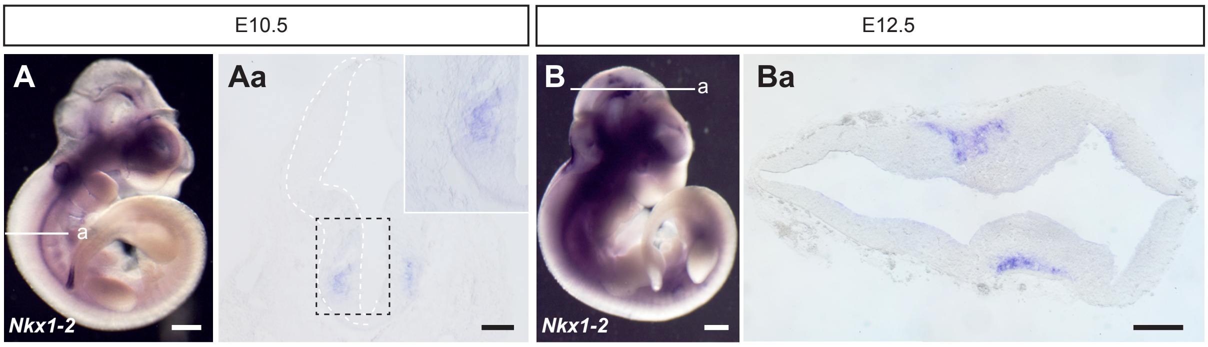

Fig. S1 *Nkx1-2* expression in neurons. Mouse embryos analysed by whole-mount *in situ* hybridization for *Nkx1-2* at (A) E10.5 and (B) E12.5. (Aa) and (Bb) show transverse sections through the regions indicated in the corresponding whole-mount embryo. Scale bar in whole-mounts, 200 μm; in transverse sections, 50 μm.

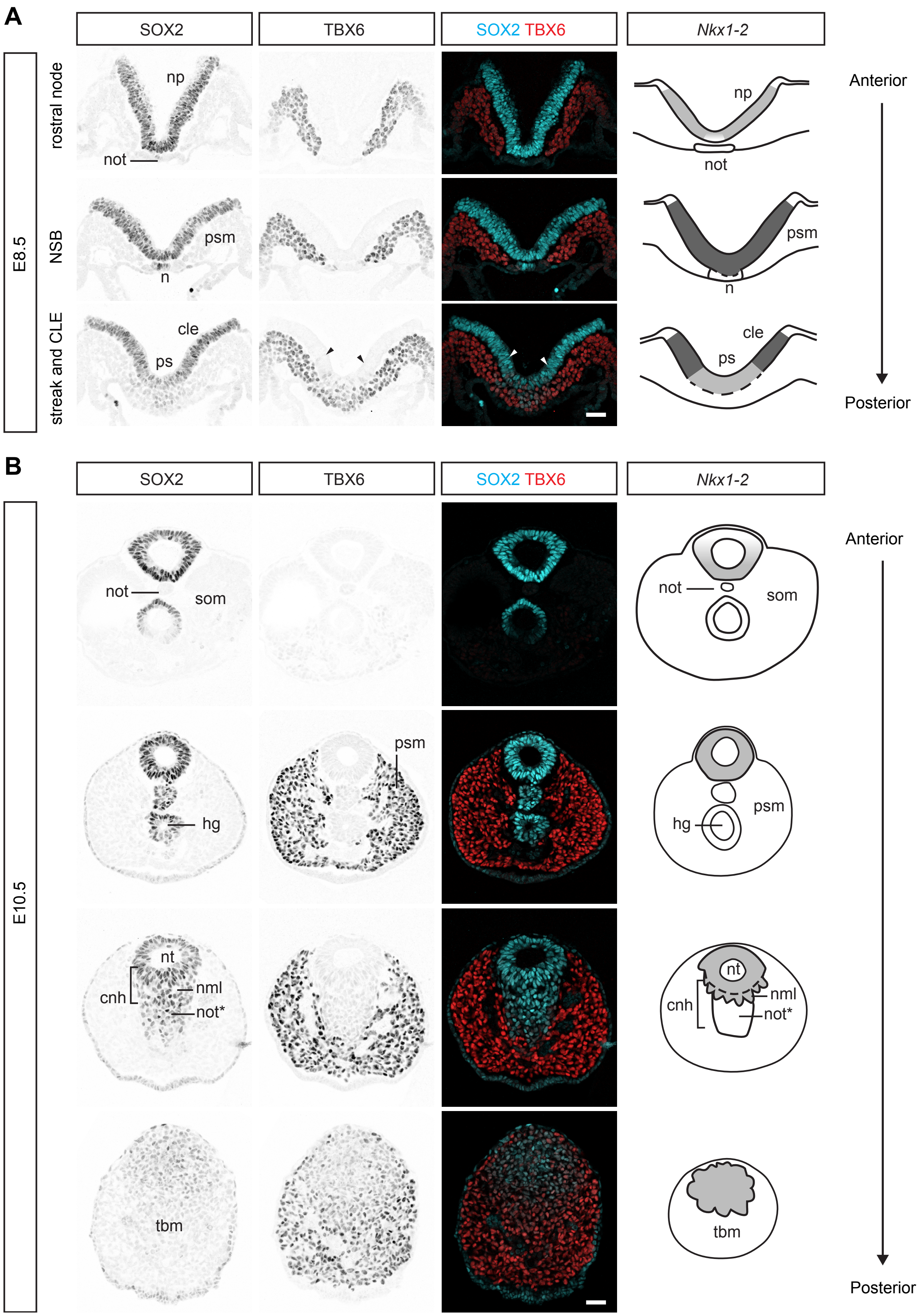

Fig. S2 SOX2 and TBX6 expression within *Nkx1-2* regions. (A) Transverse sections across the rostral node, NSB, and CLE of an E8.5 embryo immunolabelled for SOX2 and TBX6 (n=4). (B) Transverse sections across the tail end of an E10.5 embryo immunolabelled for SOX2 and TBX6 (n=4). The cartoons in (A) and (B) depict the expression pattern of *Nkx1-2* (as shown in Fig. 1). The arrowheads in (A) indicate the region that contains TBX6^{+} cells. The different levels of *Nkx1-2* expression (based on *in situ* hybridisation signal) are represented by different grey intensities (dark grey, high; light grey, low; white, no expression). The dashed lines delineate regions not limited by basement membrane. Abbreviations are the same as in Fig. 1. som, somite; nml, neuromesodermal lip. Scale bars, 50 µm.

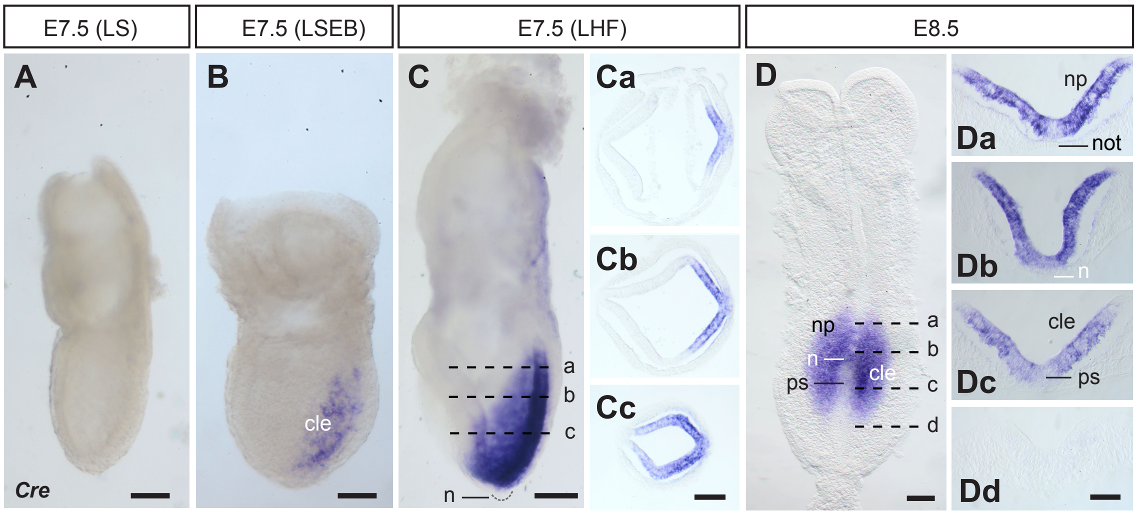

Fig. S3 CreER^{T2} expression in Nkx1-2CreER^{T2} embryos recapitulates endogenous *Nkx1-2* expression. Cre *in situ* hybridisation in whole-mount and transverse sections of Nkx1-2CreER^{T2} mouse embryos. (A) Lateral view of a late streak (LS) stage embryo. CreER^{T2} expression is undetectable at this stage. (B) Lateral view of a late streak, early allantoic bud (LSEB) stage embryo. (C) Lateral view of an early to late headfold (LHF) stage embryo. (Ca-Cc) Transverse sections through the regions indicated in C. Note that at this stage *Cre* might be expressed in a few cells in the posterior endoderm (Ca). Posterior is to the right (n=15 E7.5 embryos at different developmental stages). (D) Dorsal view of an E8.5 embryo (n=8). (Da-Dd) Transverse sections through the regions indicated in D. cle, caudal lateral epiblast; ps, primitive streak; np, neural plate; n, node; not, notochord. Scale bars, 100 µm.

Development • Supplementary information

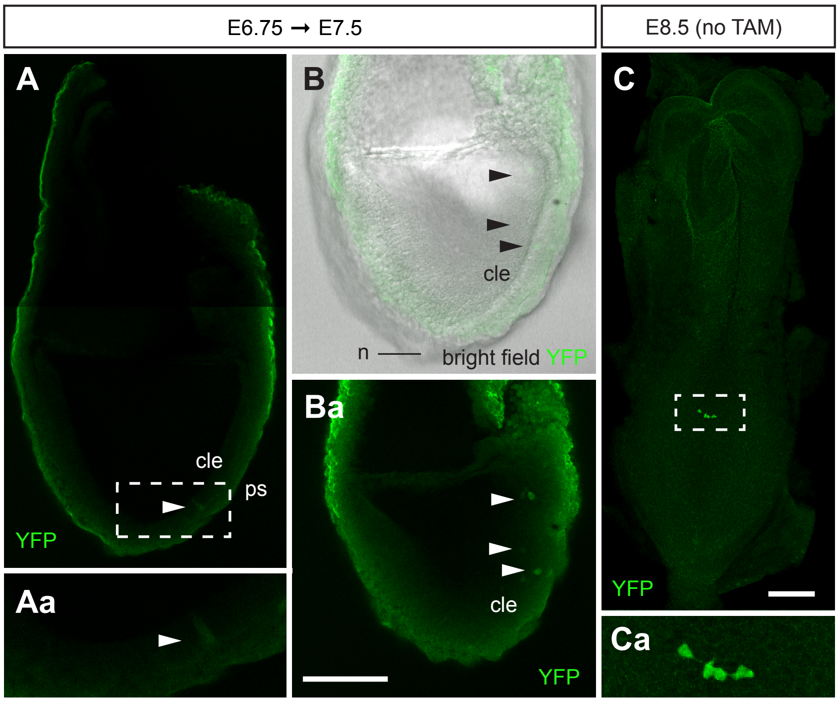

Fig. S4 Tamoxifen administration to *Nkx1-2*CreER^{T2}/YFP mice drives CreER^{T2}-mediated recombination specifically in cells within *Nkx1-2* regions. (A) and (B) Timed-pregnant Nkx1-2CreER^{T2}/YFP mice received tamoxifen at E6.75 and embryos assessed for YFP expression 18 hours later (A) Parasagittal optical section through a late bud stage Nkx1-2CreER^{T2}/YFP embryo (around E7.5) exposed to tamoxifen at E6.75 and immunolabelled for YFP on whole-mount. YFP^{+} cells were found in the NSB region and CLE which indicates that the expression of the CreER^{T2} transgene is consistent with the pattern of endogenous *Nkx1-2* expression (Figure 1) (Schubert et al., 1995) (n=6). (Aa) Higher magnification of the region indicated in A. (B) Maximum intensity projection (MIP) of four optical sections (i.e. 16 µm) of the embryo in A. YFP^{+} cells were also found more posteriorly throughout the CLE (arrowheads). (Ba) YFP channel of B. Note that even if early post-implantation embryos are highly autofluorescent (diffuse green signal in A to Ba), cells with clear cytoplasmic YFP stood out above the background signal. (C) To assess the extent of potential spontaneous recombination in Nkx1-2CreER^{T2}/YFP mice, embryos not exposed to tamoxifen (no TAM) were analysed for YFP^{+} cells on whole-mounts at E8.5. (D) MIP of an Nkx1. 2CreER^{T2}/YFP embryo at E8.5 in the absence of tamoxifen induction. All embryos analysed (n=5) showed low levels of spontaneous recombination, from 4 to 9 YFP^{+} cells each (6 ± 1 cells/embryo). The close proximity and number of YFP^{+} cells suggest that they originate from a single recombination event at around E7.5 because cells have a cell cycle of ~6-7h (Snow, 1977; Tzouanacou et al., 2009) and it takes ~4h to detect YFP (data not shown). (Ca) Higher magnification of the region indicated in C. Abbreviations are the same as in Fig. 1. Scale bars, 100 µm.

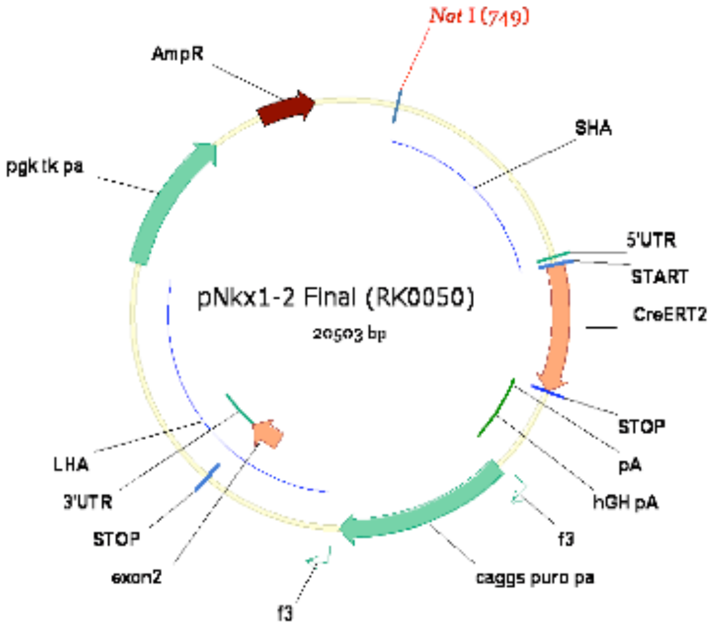

Fig. S5 Map of the targeting vector used to knock-in the CreER^{T2} cassette into the *Nkx1-2* locus. NotI was used to linearise the vector before homologous recombination. Puromycin (puro) and thymidine kinase (tk) were used as selection markers as described in Materials and Methods. SHA, short homology arm; LHA, long homology arm; pA, polyadenylation site.

Development • Supplementary information

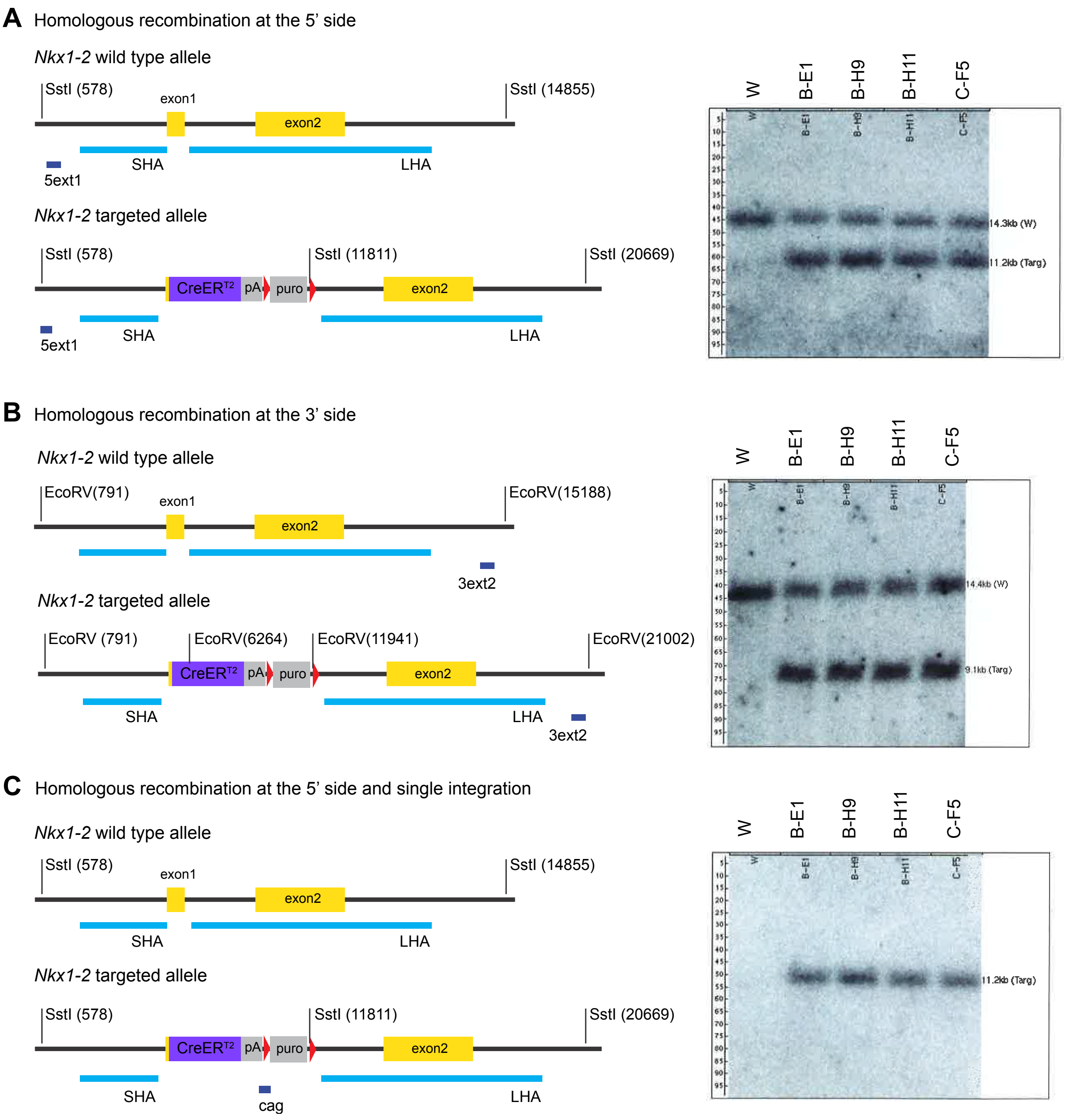

Fig. S6 Southern blot and PCR analysis confirms that targeted clones carry the CreER^{T2} cassette. Southern blot analyses of genomic DNA from wild type (W), and targeted clones B-E1, B-H9, B-H11 and C-F5. The sizes of the wild type allele (W) and the targeted allele (Targ) are shown in the Southern blot. (A) The genomic DNA was digested with the restriction enzyme SstI and a 5' probe (5ext1) was used to confirm correct homologous recombination at the 5' side. (B) The genomic DNA was digested with the restriction enzyme EcoRV and a 3' probe (3ext2) was used to confirm correct homologous recombination at the 3' side. (C) The genomic DNA was digested with SstI and a probe (cag) that detects a region located within the puro selection cassette used to confirm both correct homologous recombination at the 5' side and single integration of the cassette in all clones. The primer sequences for the PCR amplification of the external probes can be found in Table S2. The black line in the schematics represents the genomic DNA. SHA, short homology arms; LHA, long homology arms.

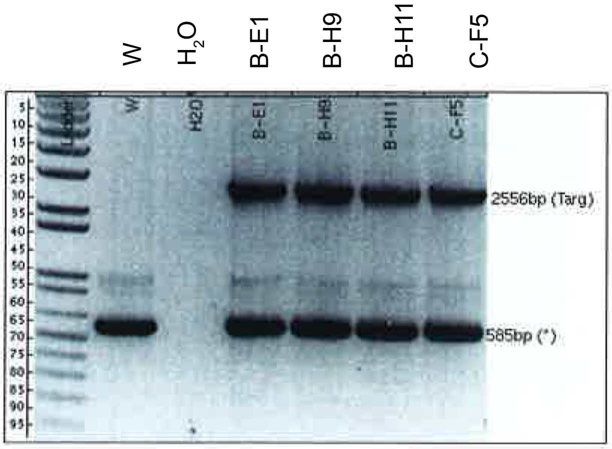

Fig. S7 PCR analysis confirms that targeted clones carry the CreER^{T2} cassette. The insertion of the CreER^{T2} cassette as detected in all targeted clones (B-E1, B-H9, B-H11, C-F5) by sequencing the 5' junction between the *Nkx1-2* and CreER^{T2} regions but not in wild type clones (W). Targ, targeted allele; *, internal control for PCR amplification. Primer sequences can be found in Table S3.

Development • Supplementary information

Data S1 *Nkx1-1* expression is undetectable in *Nkx1-2*-expressing cells.

The *Nkx1-1* gene is a paralog of *Nkx1-2* and thus both genes could carry out similar functions during embryonic development. To test this, we set out to investigate whether *Nkx1-1* is also expressed in *Nkx1-2* regions by *in situ* hybridisation. Simon and Lufkin (Simon and Lufkin, 2003) reported that *Nkx1-1* expression "can be detected as early as embryonic stage E10.5". We have tried to revisit *Nkx1-1* expression using the following strategies:

1. We used the primers described in the paper (covering the end of intron2/start of exon2) to try and clone the gene, but were unable to amplify the correct product. We think that this could be due to the high GC content of the primers (GC 80%, Tm 70+).

   Primer pair 1: mNkx1-1 forward 5'-TCCTGGGGCGGAGCGGGCAGGGCGG-3', mNkx1-1 reverse 5'-CGGCGCGCTGGTGTCAGCGCCT-3'
2. We redesigned primers with lower GC content to target a similar region (end of intron2/start of exon2), but these did not amplify the correct product either.

   Primer pair 2: mNkx1-1 forward 5'-GCAAGCAATCAAGGAGGAAC-3', mNkx1-1 reverse 5'-ACAGGTAGCGAGTGGCCTTA-3'
3. We used a new set of primers covering ~800 nucleotides of the *Nkx1-1* coding sequence (out of 1.3kb full length) on oligo dT-primed cDNA from E13.5 and E9.5 embryos. Again, the products generated were not the correct product/sequence.

   Primer pair 3: mNkx1-1 forward 5'-GGCTACAGCTCGGGACACTA-3', mNkx1-1 reverse 5'-GAGCTGCTCGTAGGTGAAGG-3'
4. We re-checked the genomic sequence of *Nkx1-1* and found that the Ensembl version misses out a bit of 5' coding sequence in the first exon. The Kozak sequence is there so it should be translated. Based on Simon and Lufkin 2003, Nkx1-1 mRNA might be more abundant at later stages. We tried again cloning the gene from E13.5 oligodT-primed cDNA, but were still unable to amplify the predicted product.

Given these difficulties we took a different approach and checked whether *Nkx1-1* is normally expressed in NMPs by interrogating two published single-cell RNA-sequencing data sets: the Koch (Koch et al., 2017) and the Gouti (Gouti et al., 2017) data sets. We found that *Nkx1-1* is undetectable in any of the 53 NMP cells of the Koch data set. Similarly, *Nkx1-1* is also undetectable in the E8.5 and E9.5 NMPs of the Gouti data set, and in their *in vitro*-derived NMPs. *Nkx1-1* expression is also effectively undetectable in bulk RNA-sequencing data of *in vitro*-derived NMPs from Gouti et al. (Gouti et al., 2014) whereas *Nkx1-2* expression is indeed detected (Fig. S8, Robert Blassberg, James Briscoe lab, The Francis Crick Institute, personal communication).

Development • Supplementary information

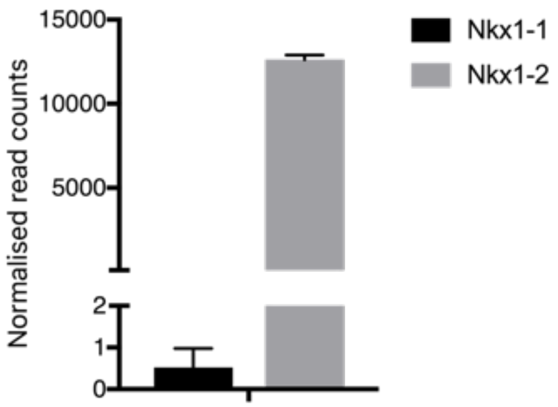

Fig. S8 Bar plot showing the relative expression of *Nkx1-1* and *Nkx1-2* generated from the bulk mRNA-sequencing data of *in vitro*-derived NMPs (data kindly provided by Robert Blassberg from (Gouti et al., 2014)). The normalised read counts for *Nkx1-1* were so low that did not pass filtering steps before downstream analysis.

Taken all together, the lack of *Nkx1-1* expression does not seem to reflect a limitation of single-cell RNA-sequencing technologies in sampling low-expressed genes; but rather, that *Nkx1-1* is not expressed in NMPs before E10.5. All in all, we have not found any evidence suggesting that *Nkx1-1* is co-expressed with *Nkx1-2* in axial progenitors. However, we still do not know whether *Nkx1-1* is upregulated to compensate for the loss of *Nkx1-2* function.

Table S1 Sequence of the targeting vector used to know-in CreER^{T2} into the *Nkx1-2* locus.

NotI Homology arms

GGGGGTTCGTGCACACAGCCCAGCTTGGAGCGAACGACCTACACCGAACTGAGATACCTACAGCGTGAGCTATGA
GAAAGCGCCACGCTTCCCGAAGGGAGAAAGGCGGACAGGTATCCGGTAAGCGGCAGGGTCGGAACAGGAGAGCGC
ACGAGGGAGCTTCCAGGGGGAAACGCCTGGTATCTTTATAGTCCTGTCGGGTTTCGCCACCTCTGACTTGAGCGT
CGATTTTTGTGATGCTCGTCAGGGGGGCGGAGCCTATGGAAAAACGCCAGCAACGCGGCCTTTTTACGGTTCCTG
GCCTTTTGCTGGCCTTTTGCTCACATGTTCTTTCCTGCGTTATCCCCTGATTCTGTGGATAACCGTATTACCGCC
TTTGAGTGAGCTGATACCGCTCGCCGCAGCCGAACGACCGAGCGCAGCGAGTCAGTGAGCGAGGAAGCGGAAGAG
CGCCCAATACGCAAACCGCCTCTCCCCGCGCGTTGGCCGATTCATTAATGCAGCTGGCACGACAGGTTTCCCGAC
TGGAAAGCGGGCAGTGAGCGCAACGCAATTAATGTGAGTTAGCTCACTCATTAGGCACCCCAGGCTTTACACTTT
ATGCTTCCGGCTCGTATGTTGTGTGGAATTGTGAGCGGATAACAATTTCACACAGGAAACAGCTATGACCATGAT
TACGCCAAGCTCGAAATTAACCCTCACTAAAGGGAACAAAAGCTGTCGAGATCTAGATATCGATGGCCATAGCGG
CCGCGGCACTCAGAGTTCACAGCCACAGGATGGGGTAAACAACAGTGCTGAGCTGTAAGTGACAGGCCAGGGATC
AGGTAAACAAACCGGTCCTGATGCTGCCGCCAGAGGCATCCTTTTAAAATTCACTGTCTTTTTTCTTCCCTCTTC
ACGGAGTGACTTGAACTGAAGAAGCACATGTTGGGGAAAGCTATCCAAGTGCCAAGTAGAGGGATGCATCCATGC
CTGTCTCCATAGTCAGTGCCTCCCTCTGGCCTTCAGCTGTGCCTGATGAAGCTGTCTCTTTGGCATGTATGTCCT
AACCTGTGGGCTGGCTCAAACAGAGAACTCCCCTTGTATTTATTTCAGGAACATATGATTTTATCTTAGGGACCT
TAATGCAGCTCATAGTATTACCCACCCTCCCTCTGCATAAACCACACATTCTTTCTCTCCATTGTTAATCCTGTT
CCCAGGGTGGGTGGCAGCTTCTCATGGCTGTGCCGGAATTACAGGCCACTTTGTTTGGACAATCGCTTGAGAAGC
CTGGCATCATTGATTTGCATCAGTGCCTCTGGAATAGCCTTTTGCCATCTCCTTAAAGATACTATCTAAGCTGTG
AAATTCATTAAATAGCCCCAGAAATGGATCAGGTTATTTAACACAACATCCCTAATGGCACAATATCTCAGCTGT
CCACACTGATAGCGAACATTGTCTTGGATAGCGTTATCAGCCCAGGACACCCGCTATGAATATTGATCCTGGGCT
TGTTACAGCCATCACTTGCCATCAGGACAGGTCAGAGTTGGAACCCACCTAGCATCAAAGCAATTTAAAGACAGC
ACTCAAATGGAATTTTAATGCCCAGGCTGGGGGGAGGGGAGAGGGGAAAAAAAAGAAAGAAAGAAAAAAAACAAC
CAAATCAACTTCGTGCACATAGGGAAGATGGCTTGCCAGATGTGAACATATGTATTATAGAGAAGCGTCCTGGAT
AACATTTGTGTCAGAACGTTAGTGAGTCAAGCACTTTTTCTCAGTTGTTCCCAAAGGGAGGTTCTGCTGCCTTAT
GGTTCGTGCAAAACAAGTAGCCAGAAAAAAAGGCTCCCTTAAGCTTCCTGCTTCCACTCCCCCATCAACAGAAGG
ACGTCTTTGGGTGTTTGCCTGTGGGCTGAGTGAGTGAAGCGGTGTTGAGAGAGAGACACATAGGTGTTTCTCCCA
TCTCAGAGTCCTGGAGGCTCATACTGCGGTGAACACACAAGAGAAATGGTGAGATGGTGAAGAGAGGAATTTAAA
ACCAGCCACACACAAGAAGGCCTGTCTACAGATCTGTAGGCTACAGTACGTGACCTCTCCTGCTTGTCCCTTTTC
TGTGTTTAGCACCATAGACTTCAAAGGAAAGATAAATCCTAGGAGAGGAAACACTTTCTATAATGTAAACAACAA
CAACAAAAGATAACTGATCCCAGTTCTGTGTGATCGTGGCTGGGGAAGGGGTCTGTCCCTCTGCTTTGGGATGTG
ATGCCCCTAGAACACTTGCTAATCCCCAGTCCTAGGAAACGACTCAATGTGTCTTCCAGAGAAAAAGGTAAGGCA
AGGAATCATGGAAAACCACAAACATTCTATGGATTCCCGAATGAGTGACCTTAGGCAGATCTTTCCCAGCTATCC
TGAAAAACCTAATATATATGGAAAAAGGAAAAAAGGAATTGGGGTTAGTAGGGGATGAGAGACAAGGAAACCGAA
GACTTATAAAAAATGTGGGAACTAGGTGAGAAACGGTTATCCAAACAGAAGACACAGTATGCGCATTACTGAAGA
ATCTCTGATTTGCACGCATTGTCCCTTGGCCTGACCTCTGGGTTGTCTTTTCCCTGAGTTTCACAAAGCGCGCAT
TCCCTTGGCAAGGGTGGGGGTCGGGGGTTGGGGGTTCTGGTGGGCTTGGTCTGGGCACTTCCTTCCCTTAGCTTT
AAAAGCTCATTCCGTCTTTACTTTAGGTCTGGAGCTGGCTTGAATTTCGGCAAGGCTTTAAAGTCGGCAGGGAAT
ATGGTGGCCACCCCTGGGAGGGGCTGAAAGGTTTGAATGAGAAAAGCTACAAGGAGCAAGCCCCCAAGGACACCT
CTTTAATAACAGATTGGATGAGCAGCTCCCAGCACTTTAGTGAGGAGTCCCCTCCTCTTCCTACTTCTTACCTGC
TTAGACAATATTAATTGTCAAGACATTGTCTGGGAACCACATGGGGCAAAGAAGAAAGAAGTGGCGGGGGGGGGG
GGGGGTTCCAAGGCGGTTCTGTGCAGAGGGGCTAATGCTTCAGAATTTGTCGCTGAAATGAATGCCTTAAAAAAA
AAAAAAGGAATATCACCGTGGGTGTGGCTATGCAGACTTTCCCTCACTGCTAATGCCGGCTGTCCTCCCTGGAGA
CTTGGGGGGCAGATGGGGCCATTTTAATCGGCTCTCATCTCTGGTCTTAAATTCAAACCACTAGGCATCTTTTAA
CCTTTTCCTTCTTCACGCTGGGAAAGGAACCCAGAGAGTCAATTTCTGATGTTAATGATAGAATCTGTGACTGAG
AAGTCCTGACAGGCAATGAAAATGGTATCCGAGGAGGGATACTCACCTTTAGCCAAGCCACCTAGCCCTAAGCCT
TCAGCTCTCTGGGCAGGCGTATTTCCATAGACCCCTTTTGACCAGGCCCTGCTGGTCTTCCGAGTGGAAAGAAAA
AACTCCCCTCCAAGAAGGTTCCCCCCCCCCCCCCGCACACACACACATATCCTAAGCCATCAGGATGACCTGAAA
AACCTTGGCCCAGTCCAGACTCTTGTCATCTGTAGCGCCACCGCTCATCTTTGAACCTCATTTTCCTTCTGTTTG
AAACCACAGACTGTGGTTCCCAGGCCACACCCACCTCACAGGGGGGAATGCCTAGAAAATCAAAAGAGAGTGAGA
AAGCCCCAGGGCTTTATAAACTGTAAATGCACAGAGGCTTTAAAATTTTTACGTAAATGTGCCCGAAGCCTTTAC
TGTGGTTCAGGGATGGGGCTTGAGATGCACCGTGCATCATGGCGAAGCAATCAGGTCTGGCCTCTGTGGCATCGC
CAGGTCAAAGGAAGATGGCAAGTGCCCCAAGAGAAATAAGTAAACTTCAGTCAGGTGAATGTGTGTGCAGATGAG
GTAGCGGAGATGGAATCCTCCCTCACGCCCTTCACGAGTCTCCGCAGCATGGAAAGATTTTCACACAGCATGCCA
CGGTGACCCGGCGAACCGGGAGCGCGCATTGGCGCGCGAGGGTCCCTCTAGTCAGCGCTCCCTCCCCCCTCCGGC
TGCCCAGGATAGTCTCGTGGAGGCTGCAAGCGCTCACTGCACGTGGAAGGGGTCTGGCCAGCTCAAGTGCCAATC
CTCAAGCAGCCCCCCCCCCCGCCCCCTTCCCCGCTTCTCTCCTCCCGCCCCGCCTCCTTGCCCCCCTCTACAGCT
TCACTTGGCGGGGAAATTCCAGTTCGCGGCTGGAGAGCTGTGGCCAAACCACTGCAGAAGACAGCGGCTGAGCGC
ATCCAGCCCCGACCCCAGTACGGCTGGCCTCGGGCCCGCGGGAGGCGGGCATGTCCAATTTACTGACCGTACACC
AAAATTTGCCTGCATTACCGGTCGATGCAACGAGTGATGAGGTTCGCAAGAACCTGATGGACATGTTCAGGGATC

Development • Supplementary information

GCCAGGCGTTTTCTGAGCATACCTGGAAAATGCTTCTGTCCGTTTGCCGGTCGTGGGCGGCATGGTGCAAGTTGA
ATAACCGGAAATGGTTTCCCGCAGAACCTGAAGATGTTCGCGATTATCTTCTATATCTTCAGGCGCGCGGTCTGG
CAGTAAAAACTATCCAGCAACATTTGGGCCAGCTAAACATGCTTCATCGTCGGTCCGGGCTGCCACGACCAAGTG
ACAGCAATGCTGTTTCACTGGTTATGCGGCGGATCCGAAAAGAAAACGTTGATGCCGGTGAACGTGCAAAACAGG
CTCTAGCGTTCGAACGCACTGATTTCGACCAGGTTCGTTCACTCATGGAAAATAGCGATCGCTGCCAGGATATAC
GTAATCTGGCATTTCTGGGGATTGCTTATAACACCCTGTTACGTATAGCCGAAATTGCCAGGATCAGGGTTAAAG
ATATCTCACGTACTGACGGTGGGAGAATGTTAATCCATATTGGCAGAACGAAAACGCTGGTTAGCACCGCAGGTG
TAGAGAAGGCACTTAGCCTGGGGGTAACTAAACTGGTCGAGCGATGGATTTCCGTCTCTGGTGTAGCTGATGATC
CGAATAACTACCTGTTTTGCCGGGTCAGAAAAAATGGTGTTGCCGCGCCATCTGCCACCAGCCAGCTATCAACTC
GCGCCCTGGAAGGGATTTTTGAAGCAACTCATCGATTGATTTACGGCGCTAAGGATGACTCTGGTCAGAGATACC
TGGCCTGGTCTGGACACAGTGCCCGTGTCGGAGCCGCGCGAGATATGGCCCGCGCTGGAGTTTCAATACCGGAGA
TCATGCAAGCTGGTGGCTGGACCAATGTAAATATTGTCATGAACTATATCCGTAACCTGGATAGTGAAACAGGGG
CAATGGTGCGCCTGCTGGAAGATGGCGATTCTGCTGGAGACATGAGAGCTGCCAACCTTTGGCCAAGCCCGCTCA
TGATCAAACGCTCTAAGAAGAACAGCCTGGCCTTGTCCCTGACGGCCGACCAGATGGTCAGTGCCTTGTTGGATG
CTGAGCCCCCCATACTCTATTCCGAGTATGATCCTACCAGACCCTTCAGTGAAGCTTCGATGATGGGCTTACTGA
CCAACCTGGCAGACAGGGAGCTGGTTCACATGATCAACTGGGCGAAGAGGGTGCCAGGCTTTGTGGATTTGACCC
TCCATGATCAGGTCCACCTTCTAGAATGTGCCTGGCTAGAGATCCTGATGATTGGTCTCGTCTGGCGCTCCATGG
AGCACCCAGTGAAGCTACTGTTTGCTCCTAACTTGCTCTTGGACAGGAACCAGGGAAAATGTGTAGAGGGCATGG
TGGAGATCTTCGACATGCTGCTGGCTACATCATCTCGGTTCCGCATGATGAATCTGCAGGGAGAGGAGTTTGTGT
GCCTCAAATCTATTATTTTGCTTAATTCTGGAGTGTACACATTTCTGTCCAGCACCCTGAAGTCTCTGGAAGAGA
AGGACCATATCCACCGAGTCCTGGACAAGATCACAGACACTTTGATCCACCTGATGGCCAAGGCAGGCCTGACCC
TGCAGCAGCAGCACCAGCGGCTGGCCCAGCTCCTCCTCATCCTCTCCCACATCAGGCACATGAGTAACAAAGGCA
TGGAGCATCTGTACAGCATGAAGTGCAAGAACGTGGTGCCCCTCTATGACCTGCTGCTGGAGGCGGCCGACGCCC
ACCGCCTACATGCGCCCACTAGCCGTGGAGGGGCATCCGTGGAGGAGACGGACCAAAGCCACTTGGCCACTGCGG
GCTCTACTTCATCGCATTCCTTGCAAAAGTATTACATCACGGGGGAGGCAGAGGGTTTCCCTGCCACAGTCTGAG
CGGCCGACCGGTTCGAGATCCAGGCGCGGATCAATAAAAGATCATTATTTTCAATAGATCTGTGTGTTGGTTTTT
TGTGTGCCTTGGGGGAGGGGGAGGCCAGAATGAGGCGCGGCCAAGGGGGAGGGGGAGGCCAGAATGACCTTGGGG
GAGGGGGAGGCCAGAATGACCTTGGGGGAGGGGGAGGCCAGAATGAGGCGCGACCTAGGAGCAATTGATGGCGCG
CCATCCAGGCGCGGATCAATAAAAGATCATTATTTTCAATAGATCTGTGTGTTGGTTTTTTGTGTGCCTTGGGGG
AGGGGGAGGCCAGAATGAGGCGCGGCCAAGGGGGAGGGGGAGGCCAGAATGACCTTGGGGGAGGGGGAGGCCAGA
ATGACCTTGGGGGAGGGGGAGGCCAGAATGAGGCGCGATCCGTCGACTTAATTAAGGCCAGGGATCTTCAAGCAG
ACCTACAGCAAGTTCGACACAAACTCACACAACGATGACGCACTACTCAAGAACTACGGGCTGCTCTACTGCTTC
AGGAAGGACATGGACAAGGTCGAGACATTCCTGCGCATCGTGCAGTGCCGCTCTGTGGAGGGCAGCTGTGGCTTC
TAGCTGCCCGGGTGGCATCCCTGTGACCCCTCCCCAGTGCCTCTCCTGGCCCTGGAAGTTGCCACTCCAGTGCCC
ACCAGCCTTGTCCTAATAAAATTAAGTTGCATCATTTTGTCTGACTAGGTGTCCTTCTATAATATTATGGGGTGG
AGGGGGGTGGTATGGAGCAAGGGGCAAGTTGGGAAGAAAACCTGTAGGGCCTGCGGGGTCTATTGGGAACCAAGC
TGGAGTGCAGTGGCACAATCTTGGCTCACTGCAATCTCCGCCTCCTGGGTTCAAGCGATTCTCCTGCCTCAGCCT
CCCGAGTTGTTGGGATTCCAGGCATGCATGACCAGGCTCAGCTAATTTTTGTTTTTTTGGTAGAGACGGGGTTTT
ACCATATTGGCCAGGCTGGTCTCCAACTCCTAATCTCAGGTGATCTACCCACCTTGGCCTCCCAAATTGCTGGGA
TTACAGGCGTGAACCACTGCTCCCTTCCCTGTCCTTCTGATTTTAAAATAACTATACCAGCAGGAGGACGTCCAG
ACACAGCATAGGCTACCTGGCCATGCCCAACCGGTGGGACATTTGAGTTGCTTGCTTGGCACTGTCCTCTCATGC
GTTGGGTCCACTCAGTAGATGCCTGTTGAATTAAGGGCGCGCCGTTACGCTAGGGATAACAGGCTCGACTCGAGC
CTAGGGGTAACCATTTAAATGAAGTTCCTATTCCGAAGTTCCTATTCTTCAAATAGTATAGGAACTTCTCGGGGC
TAGCTTGCATGCCTGCAGGTTTTCGACATTGATTATTGACTAGTTATTAATAGTAATCAATTACGGGGTCATTAG
TTCATAGCCCATATATGGAGTTCCGCGTTACATAACTTACGGTAAATGGCCCGCCTGGCTGACCGCCCAACGACC
CCCGCCCATTGACGTCAATAATGACGTATGTTCCCATAGTAACGCCAATAGGGACTTTCCATTGACGTCAATGGG
TGGAGTATTTACGGTAAACTGCCCACTTGGCAGTACATCAAGTGTATCATATGCCAAGTACGCCCCCTATTGACG
TCAATGACGGTAAATGGCCCGCCTGGCATTATGCCCAGTACATGACCTTATGGGACTTTCCTACTTGGCAGTACA
TCTACGTATTAGTCATCGCTATTACCATGGTCGAGGTGAGCCCCACGTTCTGCTTCACTCTCCCCATCTCCCCCC
CCTCCCCACCCCCAATTTTGTATTTATTTATTTTTTAATTATTTTGTGCAGCGATGGGGGCGGGGGGGGGGGGG
CGCGCGCCAGGCGGGGCGGGGCGGGGCGAGGGGCGGGGCGGGGCGAGGCGGAGAGGTGCGGCGGCAGCCAATCAG
AGCGGCGCGCTCCGAAAGTTTCCTTTTATGGCGAGGCGGCGGCGGCGGCGGCCCTATAAAAAGCGAAGCGCGCGG
CGGGCGGGAGTCGCTGCGTTGCCTTCGCCCCGTGCCCCGCTCCGCGCCGCCTCGCGCCGCCCGCCCCGGCTCTGA
CTGACCGCGTTACTCCCACAGGTGAGCGGGCGGGACGGCCCTTCTCCTCCGGGCTGTAATTAGCGCTTGGTTTAA
TGACGGCTCGTTTCTTTTCTGTGGCTGCGTGAAAGCCTTAAAGGGCTCCGGGAGGGCCCTTTGTGCGGGGGGGAG
CGGCTCGGGGGGTGCGTGCGTGTGTGTGTGCGTGGGGAGCGCCGCGTGCGGCCCGCGCTGCCCGGCGGCTGTGAG
CGCTGCGGGCGCGGCGCGGGGCTTTGTGCGCTCCGCGTGTGCGCGAGGGGAGCGCGGCCGGGGGCGGTGCCCCGC
GGTGCGGGGGGGCTGCGAGGGGAACAAAGGCTGCGTGCGGGGTGTGTGCGTGGGGGGGTGAGCAGGGGGTGTGGG
CGCGGCGGTCGGGCTGTAACCCCCCCCTGCACCCCCCTCCCCGAGTTGCTGAGCACGGCCCGGCTTCGGGTGCGG
GGCTCCGTACGGGGCGTGGCGCGGGGCTCGCCGTGCCGGGCGGGGGGTGGCGGCAGGTGGGGGTGCCGGGCGGGG
CGGGGCCGCCTCGGGCCGGGGAGGGCTCGGGGGAGGGGCGCGGCGGCCCCCGGAGCGCCGGCGGCTGTCGAGGCG
CGGCGAGCCGCAGCCATTGCCTTTTATGGTAATCGTGCGAGAGGGCGCAGGGACTTCCTTTGTCCCAAATCTGTG
CGGAGCCGAAATCTGGGAGGCGCCGCCGCACCCCCTCTAGCGGGCGCGGGGCGAAGCGGTGCGGCGCCGGCAGGA

Development • Supplementary information

AGGAAATGGGCGGGGAGGGCCTTCGTGCGTCGCCGCGCCGCCGTCCCCTTCTCCCTCTCCAGCCTCGGGGCTGTC
CGCGGGGGGACGGCTGCCTTCGGGGGGGACGGGGCAGGGCGGGGTTCGGCTTCTGGCGTGTGACCGGCGGCTCTA
GAAGCGTTGGGGTGAGTACTCCCTCTCAAAAGCGGGCATGACTTCTGCGCTAAGATTGTCAGTTTCCAAAAACGA
GGAGGATTTGATATTCACCTGGCCCGCGGTGATGCCTTTGAGGGTGGCCGCGTCCATCTGGTCAGAAAAGACAAT
CTTTTTGTTGTCAAGCTTGAGGTGTGGCAGGCTTGAGATCTGGCCATACACTTGAGTGACATTGACATCCACTTT
GCCTTTCTCTCCACAGGTGTCCACTCCCAGGGCGGCCTCCGGAGCGATCGCCGATCCGCCTAGCCTTCCATGACC
GAGTACAAGCCCACGGTGCGCCTCGCCACCCGCGACGACGTCCCCCGGGCCGTACGCACCCTCGCCGCCGCGTTC
GCCGACTACCCCGCCACGCGCCACACCGTCGACCCGGACCGCCACATCGAGCGGGTCACCGAGCTGCAAGAACTC
TTCCTCACGCGCGTCGGGCTCGACATCGGCAAGGTGTGGGTCGCGGACGACGGCGCCGCGGTGGCGGTCTGGACC
ACGCCGGAGAGCGTCGAAGCGGGGGCGGTGTTCGCCGAGATCGGCCCGCGCATGGCCGAGTTGAGCGGTTCCCGG
CTGGCCGCGCAGCAACAGATGGAAGGCCTCCTGGCGCCGCACCGGCCCAAGGAGCCCGCGTGGTTCCTGGCCACC
GTCGGCGTCTCGCCCGACCACCAGGGCAAGGGTCTGGGCAGCGCCGTCGTGCTCCCCGGAGTGGAGGCGGCCGAG
CGCGCCGGGGTGCCCGCCTTCCTGGAGACCTCCGCGCCCCGCAACCTCCCCTTCTACGAGCGGCTCGGCTTCACC
GTCACCGCCGACGTCGAGGTGCCCGAAGGACCGCGCACCTGGTGCATGACCCGCAAGCCCGGTGCCTGAACCGGT
TCGAGATCCAGGCGCGGATCAATAAAAGATCATTATTTTCAATAGATCTGTGTGTTGGTTTTTTGTGTGCCTTGG
GGGAGGGGGAGGCCAGAATGAGGCGCGGCCAAGGGGGAGGGGGAGGCCAGAATGACCTTGGGGGAGGGGGAGGCC
AGAATGACCTTGGGGGAGGGGGAGGCCAGAATGAGGCGCGTATCCTTAGGCAGGAATATTCATGATCACGAGCTC
CAGCTGCTGGACATGTTGTCGATCCTGCAGGACGCGTCAATTGCTAGCGGTACCGAAGTTCCTATTCCGAAGTTC
CTATTCTTCAAATAGTATAGGAACTTCATTTAAATGGCGCGCCTTAATTAAGATATCTTTCCTGAACACCTGCTC
AGTGAATGAGCGCCACAACTCTGACTGGCTCACATAAACTACAGCTCAGGCTCACTGATCTAATGGAAGTGTATT
GTGCTCTCCACGTGCTAGAACCTGGGCGAGGAGCTTGGGATAAAGGTTATGAAAATACCTTCCAACTATGTAGTG
TCATTAACACCACTCCCATTTTATGTATGGGGCAACCGAGGCTCGGGCCTTCCCCGGATTAGGTGCTTTTTTTTC
AACCACTCGGAAACATCTTTAGAGCCCATGCTCTTTCCCGGACTCAACAGGGGACGCCCCCACCCCCAATAGCTT
TTGGTGTTCTCTTCTAGACCTTTCCCCCTTATCCCTGAAGTTGGTGGACACAAAAGAAAGGGTTCCGGAAAGTTT
GTGGCTGGAAGAACTTAAGATAGCAGGATTCATGCTTAGTGGGACATGAGCATGGGATTGATTATCTGGGGAGAC
GCTGAAGGTCTAGGGATTCAGGTCCCAGGGAGGGCTGTATATAGCACAGCCCCTCCTCCTCCTTCCTAAGTTTGG
AGGTGATTGTCTGGGGCCCCCGGAAGCCTCTGGTGGCCTGGCTTGGGTCCCGGCCGCGGGCCTGGATTAATTGTT
TGGATGGTCTCTCTGTGGGGCCGCGCTCCCCTCCCCCTAACTCCTTGATCGATATTCTATTACTGGCGCCTGCAT
ATCTCCTGCCCGCAGCTTGCAGGTACTGACTTCAAACCCCCTTTCCCTGTCTGATCCTAATATTGTTCGATTAAA
ACTTACCTTTAATAGATGACATCTATCGATCAGGCCCCGGACAAATCTGAAACTCTATTAACACTACTGGAGAGA
GAAGATGGGAAAGGGGGAATTTCTCTTTTTTGGGGGGATGGGGGTTTAATAACTTCTTTTTTAATTGCTAGGAGT
TGCACAGCAACATAGGCTCCTAGGGACTAGAGAGATGCACTAGTAAACGCACTTGACATTCCCTATCATAATGCA
CAGCCGACTTTGCTCTTCCCGGTTTGATAAAGTCACTATACTTTCTTGAAACGGGGTGTCATCCAACATGTACTT
CTTGTGTGCCTACTTACTGGCAGATTTGTAAAGGACACTGTACTCCCGAATCTGCTCTCTAGGGTATGTGGATTC
AGAAACCAAGAGGGCAAAGTCGGGAGAGGGGAGGAACTGGGAGTTAGATCAAGGGCAAATCTGAGTGCTCTTGGG
AGGACAAGAAGGTTTGGGAGTGCCATTGGGTAGAATTGTTATGGGCTTCTGGTCACCATCCCCTTCTCTTTTAGA
TGCGGTGGGCCGAGGCATTGACCCCGGGTCTCCGGTGGAGGGCTCCGAGGCAGAGGAGGAGGAGGAAGCTGAGGA
TGCGGGGCGCGCGCACCAGCCAGAGCGCTGGCAGGGGGTCCATGAGGGCTCACCAGAGGCCCGGGCAGTAGCGGT
GGGAACGGAAGAGAGCGGCGCGGAAGGCCTCCCGGCGTCCCCGGGCTCTCCCGGTTCCCCGCGGCCCCGACGCCG
GCGAGCCGAGTCCAGCTGCGCCAAGCCAAGGCGCGCGCGCACTGCCTTCACTTACGAGCAGCTGGTGGCCCTAGA
GAACAAGTTTCGAGCCACGCGCTATCTGTCGGTGTGCGAACGCCTGAACCTGGCGCTGTCGCTCAGCCTCACGGA
GACGCAGGTCAAAATTTGGTTCCAGAACCGCAGGACCAAGTGGAAGAAGCAGAACCCAGGGGCCGACGGCGCCGT
GCAGGCGGGAGGCGGTGCGCCTCAGCCTGGGACACCGGGCGCGGTAGCGGGGGGCGGTGGCAGTGCCACTGGGAG
TAGTCCTGGCCCTCCGGTTCCTGGCGCTCTGCCCTATCAGACTTTCCCCACCTATCCTGCCACCAACGTCCTCTT
CCCTGCCGCCTCCTTCCCGCTGACGACCGCCGCCAACGGGAGTCCCTTCACTCCATTCCTTGGGCCCTCCTACTT
GACCCCTTTCTATGCTCCACACCTATGAATCCGGTACTGCTGCTCAGTCAGAATTCACGAGTGATCAGTATGATG
ACGCGGGTCCCCGCTTCACAGAGGGGCACTGACCGCCCCCTCCTGGTGTGCCCCTTCCCTCCGACTGCTGGTGAC
GCCACCAGAGGGCGAGGAGAAGTCACCTGCAGACTACAACCACACAAGCCACTGAGGGGTCGAGGCGATGTCCGT
CCAGACCAGGCTGTTTTAAACGTTTCCAGCTTCTGGACCTCATTCTTCCCTTTGATTTACTTCGAGGGCTCTGTT
CTTTACCGTGTCTGGCTAATAAGGGTTCCCAGGATGGAGACCAGAGGGAGCCAAGATGCCCTGGGCACCAGTTGC
TAGCACCAAGGACTGAATCTTTAAAGGTGGCAGGACTTATGAAGAGGAAGAAACTTTTCTTCCTGGCGTTGGAAG
TGGCTCAGCACGGAACTCCCAAATCCTGAACAAAGCTGGAGCACGAGGAACCCAAAGCAGATGCAGTTGTTGAGT
GGGCTGGTTTCAAGATGCACGACCTTAGTGTGTGTGTGTGTGTGTGTGTGTGTGTGTGTGTAGACTCCATTGTTG
AGTGGGCTGGTTTCAAGATGCACGACCTTAGTGTGTGTGTGTGTGTGTGTGTAGACTCCATTTGTGGCCCGAAGT
TGTTCAGCGCTGTACCTATATTCACCCAGAGAAGACACGGGGCAACTCTGTTACCTCACAGGTTTTGAGAACAGC
TTGGACAGACCACAGCGGTGTCCTAAAATGCACAAAAAGGACAGAAAAGGGAGCGATGACCCAAAGGCTCTCCTG
AAGTCTGTGTGTGAGTCTTTGTGATGTACAGTGCTTGGCGCCCATCTGTCCGTTCTGCTACACTTTCACGGACTT
CAAACTGACAAGCTGGAGCAAAGCAAGGCCCAGATGCAGACATTGCCGTAAATTTCTGAAAGCACAAGCATAACA
TATTTAGATAACCTGAGATTCTGACTTGAACACAATTCTTGTTCTATATCAATTTTTGCCCCCTTTCATGATGGG
AAGAAGCAAAAGTTGTAAGGGGCGGGGAAAGAAATACTTGCCAAGCGTAAGGCTAACGTTATTGTGGAGGCCAGA
ACCTTATCAGGAATGGGCACAAGGGTACATATCTTCTAACTGCTGCTTTCTCTGTTAAAACAAAGCAAAAGTACT
CTATAAAGTTTCCCCCTCCCCCAGCCCCTGTTTGGTTTGAAGTACTCACCAGTTCCTGTGTCATTTAGTCTTGTA
GTCACACACCTCAGAGACCCAGGTGACCCCATCTACCCTAGTTTGTGTTTCATCCTAACTATTTTAGTGGTTTGA

Development • Supplementary information

TGGCAGTCAATGCCTAAATGAATAATATATTTCTTATTATTATTCCTTGGCGACCCGTACCATTAAAAATTAAAC
ATGCCAGGCGATGGAGGTTCATGCCTTTTAATCCCAGCATTTGGGAGGCAGAGGCAGGTGGATCTGGTGAATTTG
AGGCCAGCCTGGTCTTCAGAGCTGGTTTTAGGACATCCAGGGCTACACAAAGAAACCCTGTCTCGCAAAACACAA
AACAAACAAACAAAATTAAACCTAATTATCAGCAGCTTTAAGTTAAACATACACATTGCCATTGTGAACATTGTA
GTCTTGAGACAATTCACTTTCCTATTATGAGGGATGATAGGAAAATAACCCTCCTTCCCTCTCCCTTAAATGAAG
CTTTCATGTTCTGGGGCTGTAAGATTTCTGAGTATGAATGACATCATTGTATAACTGATACTTAGAGTCTTTTTC
TTCTATTCTGAAGAATAATTTCTCTGAGCACTGTATTGTAACTTCTCAAAGGACAGCTGGTGCCAGATGAGATTA
GAAATGAAGTCATTACATAGGGACCATGGTTTTATTCACGGTGTATGACACGGTGTAAACACTCCTTCCGTGACT
ACTTGTTCTCAGCAACAGGATCCATATCTGTGAACAGAGAACTTCAAATACGCAAAGATCTGAACAAAGAGGTCA
AAGGCCTGTACTAAGTGGGAAAGGTACCCTCTCAGGTACCAGAATCACGGGTGTCAGAGGAGGTGGAGACTCAGC
CTTGTGCTGTCATTACGGAGACCCTTCCTTATTCCACACATTGCCAGAGCGATGCTGGAAATGGGTGACTTTGGA
CTTATTACAAGGCCCAGTCACGTTTCAACTAACTCCTTAGATTCGTTTCTATTGATGTGGTGGCCTAATAAATTC
TTTTTTTGTAATCATTTTTGACGAATTATTCATCATTACTAGCAAAACTTTTTAACAAATTTCATAATCATCAGA
GAATATGTGGTAGTGGTGGTGGTGGTAGTGGTGGTGGTTGTGTGTGTGTGTGTGCGCGCGTGTATGCATGCTCCA
CTTCTCCTACTAGCTAATACAGCTAATGACAACAATACTTCTTTACAGAGCCACACTAGATGTATTTTTGTAAGT
AATTTTACAGATACATTCATGGCTGTTCTAGCAAAAGTAAAGCATCTTTGAAAAATTTTAATTTCTGGTCTACTC
TCTAAATCAGTATCTGAGCACTAGTGTCCCAGCCTTACTTATCAAGTTCCTAATCCTCTGCCCCCACCCCAAGAG
CCTGATAGACACCTCTTAAAAGCTTAACCATTCATTGGCCATAGATGTCTATGGGGAAAAGTGAGAGTTTTTGGT
TCTGTCTAGAAGCCGTATAACCTGAGAGCCTTTTTAAAGAGAGCGAATAAGGAACACTTCTGTGAAGAGAATCCC
AGTCCACCCCCACCCCACCCCACCCTTTTTTTTCTCATTTTGTAGGAAGGAGATGAAAGTCTCACTATGGGAGAT
TTGCTCTCTGTGTAAACCAGGCAGGCTAGGACTCACAGAGATTTTCAATCTGTGTGCCTGCGCTTTTATAGACAG
TTCGTCAACCAGGCTTGGTAGCGTACACCTGTAATTCTAGCGTTTGGGAGGCTGAAGTAGGAGGTCAGCATGAGT
TCAAATTTAGGCTGGGCTGTTTCAGGCCGTCTTAGGCTTCAGAGTTAGACCTGTCCCCAAAAGAAACAATAAAAA
CCAACTCTCAACTGCCTAGGGTTTACTGCTGTTGGGAGAGAAACATCAGAATTTCAGTAAGGGCTGGATATACTG
AAGAGTGGGCGTTGGCAGTACACAGTCTGTCACACAGATTCAGTGAGAAACACAGCTCCTCAGAACTAAGGAAAG
CTTGGTAGGCAGCCAAGAAGACACAAGTTCCCTCAGGCTCAGGTGGGAAAGCATAATTTAAACCATGGCCTCTGA
CCCCAGGATTTAAGGCAACCTGGTCCTCCATGGAAACATAACTTTGACAAATTGGTGTCTGTGCCTCTGTTTCCT
CAGGAAAATGTTTCTCACAAAAGGTAATCTTGGGAATTCATATGGGAAGCACTTAGTTTAGGACTTGATAGAACA
GTGTCATTCTAGCTGAAACAGACTTAAGAATTTTTGGACCTAGGGGGCCGGCCAAGCTTAAGGAATTCGCTAGCA
TGCATCGTCGACGAACTCCTCAGCTTTCGCGCGCGAATGGATCCTTAATTAATGTACAGGGTCCCGTTTAAACAG
TAACGCTAGGGATAACAGGGTAATATAATCGAGCTGCAGGATTCGAGGGCCCCGGCAGGTCAATTCTACCGGGTA
GGGGAGGCGCTTTTCCCAAGGCAGTCTGGAGCATGCGCTTTAGCAGCCCCGCTGGGCACTTGGCGCTACACAAGT
GGCCTCTGGCCTCGCACACATTCCACATCCACCGGTAGGCGCCAACCGGCTCCGTTCTTTGGTGGCCCCTTCGCG
CCACCTTCTACTCCTCCCCTAGTCAGGAAGTTCCCCCCCGCCCCGCAGCTCGCGTCGTGCAGGACGTGACAAATG
GAAGTAGCACGTCTCACTAGTCTCGTGCAGATGGACAGCACCGCTGAGCAATGGAAGCGGGTAGGCCTTTGGGGC
AGCGGCCAATAGCAGCTTTGCTCCTTCGCTTTCTGGGCTCAGAGGCTGGGAAGGGGTGGGTCCGGGGGCGGGCTC
AGGGGCGGGCTCAGGGGCGGGGCGGGCGCCCGAAGGTCCTCCGGAGGCCCGGCATTCTGCACGCTTCAAAAGCGC
ACGTCTGCCGCGCTGTTCTCCTCTTCCTCATCTCCGGGCCTTTCGACCTGCAGCCAATGCACCGTCCTTGCCATC
ATGGCCTCGTACCCCGGCCATCAACACGCGTCTGCGTTCGACCAGGCTGCGCGTTCTCGCGGCCATAGCAACCGA
CGTACGGCGTTGCGCCCTCGCCGGCAGCAAGAAGCCACGGAAGTCCGCCCGGAGCAGAAAATGCCCACGCTACTG
CGGGTTTATATAGACGGTCCCCACGGGATGGGGAAAACCACCACCACGCAACTGCTGGTGGCCCTGGGTTCGCGC
GACGATATCGTCTACGTACCCGAGCCGATGACTTACTGGCGGGTGCTGGGGGCTTCCGAGACAATCGCGAACATC
TACACCACACAACACCGCCTCGACCAGGGTGAGATATCGGCCGGGGACGCGGCGGTGGTAATGACAAGCGCCCAG
ATAACAATGGGCATGCCTTATGCCGTGACCGACGCCGTTCTGGCTCCTCATATCGGGGGGGAGGCTGGGAGCTCA
CATGCCCCGCCCCCGGCCCTCACCCTCATCTTCGACCGCCATCCCATCGCCGCCCTCCTGTGCTACCCGGCCGCG
CGGTACCTTATGGGCAGCATGACCCCCCAGGCCGTGCTGGCGTTCGTGGCCCTCATCCCGCCGACCTTGCCCGGC
ACCAACATCGTGCTTGGGGCCCTTCCGGAGGACAGACACATCGACCGCCTGGCCAAACGCCAGCGCCCCGGCGAG
CGGCTGGACCTGGCTATGCTGGCTGCGATTCGCCGCGTTTACGGGCTACTTGCCAATACGGTGCGGTATCTGCAG
TGCGGCGGGTCGTGGCGGGAGGACTGGGGACAGCTTTCGGGGACGGCCGTGCCGCCCCAGGGTGCCGAGCCCCAG
AGCAACGCGGGCCCACGACCCCATATCGGGGACACGTTATTTACCCTGTTTCGGGCCCCCGAGTTGCTGGCCCCC
AACGGCGACCTGTATAACGTGTTTGCCTGGGCCTTGGACGTCTTGGCCAAACGCCTCCGTTCCATGCACGTCTTT
ATCCTGGATTACGACCAATCGCCCGCCGGCTGCCGGGACGCCCTGCTGCAACTTACCTCCGGGATGGTCCAGACC
CACGTCACCACCCCCGGCTCCATACCGACGATATGCGACCTGGCGCGCACGTTTGCCCGGGAGATGGGGGAGGCT
AACTGAGGGGATCGATCCGTCCTGTAAGTCTGCAGAAATTGATGATCTATTAAACAATAAAGATGTCCACTAAAA
TGGAAGTTTTTCCTGTCATACTTTGTTAAGAAGGGTGAGAACAGAGTACCTACATTTTGAATGGAAGGATTGGAG
CTACGGGGGTGGGGGTGGGGTGGGATTAGATAAATGCCTGCTCTTTACTGAAGGCTCTTTACTATTGCTTTATGA
TAATGTTTCATAGTTGGATATCATAATTTAAACAAGCAAAACCAAATTAAGGGCCAGCTCATTCCTCCCACTCAT
GATCTATAGATCTATAGATCTCTCGTGGGATCATTGTTTTTCTCTTGATTCCCACTTTGTGGTTCTAAGTACTGT
GGTTTCCAAATGTGTCAGTTTCATAGCCTGAAGAACGAGATCAGCAGCCTCTGTTCCACATACACTTCATTCTCA
GTATTGTTTTGCCAAGTTCTAATTCCATCAGAAGCTGACTCTAGGCCGGACGCCCGGGCGACCGGCCGAGCTCCA
ATTCGCCCTATAGTGAGTCGTATTACAATTCACTGGCCGTCGTTTTACAACGTCGTGACTGGGAAAACCCTGGCG
TTACCCAACTTAATCGCCTTGCAGCACATCCCCCTTTCGCCAGCTGGCGTAATAGCGAAGAGGCCCGCACCGATC
GCCCTTCCCAACAGTTGCGCAGCCTGAATGGCGAATGGACGCGCCCTGTAGCGGCGCATTAAGCGCGGCGGGTG

Development • Supplementary information

TGGTGGTTACGCGCAGCGTGACCGCTACACTTGCCAGCGCCCTAGCGCCCGCTCCTTTCGCTTTCTTCCCTTCCT
TTCTCGCCACGTTCGCCGGCTTTCCCCGTCAAGCTCTAAATCGGGGGCTCCCTTTAGGGTTCCGATTTAGTGCTT
TACGGCACCTCGACCCCAAAAAACTTGATTAGGGTGATGGTTCACGTAGTGGGCCATCGCCCTGATAGACGGTTT
TTCGCCCTTTGACGTTGGAGTCCACGTTCTTTAATAGTGGACTCTTGTTCCAAACTGGAACAACACTCAACCCTA
TCTCGGTCTATTCTTTTGATTTATAAGGGATTTTGCCGATTTCGGCCTATTGGTTAAAAAATGAGCTGATTTAAC
AAAAATTTAACGCGAATTTTAACAAAATATTAACGCTTACAATTTAGGTGGCACTTTTCGGGGAAATGTGCGCGG
AACCCCTATTTGTTTATTTTTCTAAATACATTCAAATATGTATCCGCTCATGAGACAATAACCCTGATAAATGCT
TCAATAATATTGAAAAAGGAAGAGTATGAGTATTCAACATTTCCGTGTCGCCCTTATTCCCTTTTTTGCGGCATT
TTGCCTTCCTGTTTTTGCTCACCCAGAAACGCTGGTGAAAGTAAAAGATGCTGAAGATCAGTTGGGTGCACGAGT
GGGTTACATCGAACTGGATCTCAACAGCGGTAAGATCCTTGAGAGTTTTCGCCCCGAAGAACGTTTTCCAATGAT
GAGCACTTTTAAAGTTCTGCTATGTGGCGCGGTATTATCCCGTATTGACGCCGGGCAAGAGCAACTCGGTCGCCG
CATACACTATTCTCAGAATGACTTGGTTGAGTACTCACCAGTCACAGAAAAGCATCTTACGGATGGCATGACAGT
AAGAGAATTATGCAGTGCTGCCATAACCATGAGTGATAACACTGCGGCCAACTTACTTCTGACAACGATCGGAGG
ACCGAAGGAGCTAACCGCTTTTTTGCACAACATGGGGGATCATGTAACTCGCCTTGATCGTTGGGAACCGGAGCT
GAATGAAGCCATACCAAACGACGAGCGTGACACCACGATGCCTGTAGCAATGGCAACAACGTTGCGCAAACTATT
AACTGGCGAACTACTTACTCTAGCTTCCCGGCAACAATTAATAGACTGGATGGAGGCGGATAAAGTTGCAGGACC
ACTTCTGCGCTCGGCCCTTCCGGCTGGCTGGTTTATTGCTGATAAATCTGGAGCCGGTGAGCGTGGGTCTCGCGG
TATCATTGCAGCACTGGGGCCAGATGGTAAGCCCTCCCGTATCGTAGTTATCTACACGACGGGGAGTCAGGCAAC
TATGGATGAACGAAATAGACAGATCGCTGAGATAGGTGCCTCACTGATTAAGCATTGGTAACTGTCAGACCAAGT
TTACTCATATATACTTTAGATTGATTTAAAACTTCATTTTTAATTTAAAAGGATCTAGGTGAAGATCCTTTTTGA
TAATCTCATGACCAAAATCCCTTAACGTGAGTTTTCGTTCCACTGAGCGTCAGACCCCGTAGAAAAGATCAAAGG
ATCTTCTTGAGATCCTTTTTTTCTGCGCGTAATCTGCTGCTTGCAAACAAAAAAACCACCGCTACCAGCGGTGGT
TTGTTTGCCGGATCAAGAGCTACCAACTCTTTTTCCGAAGGTAACTGGCTTCAGCAGAGCGCAGATACCAAATAC
TGTCCTTCTAGTGTAGCCGTAGTTAGGCCACCACTTCAAGAACTCTGTAGCACCGCCTACATACCTCGCTCTGCT
AATCCTGTTACCAGTGGCTGCTGCCAGTGGCGATAAGTCGTGTCTTACCGGGTTGGACTCAAGACGATAGTTACC
GGATAAGGCGCAGCGGTCGGGCTGAACG

Development • Supplementary information

Table S2 Primer sequences for the amplification of the probes for Southern blot analysis.

Probe	Forward primer	Reverse primer
5ext1	GGTGGTTCAGCTTACTTGCTAGAGC	TCAAATCAAGCTTCAAAATCCC
3ext2	TAAACTGAGTCTTTGACAGGACATG	TCCCATGCACGCTAGGCAGGCTTTC

Table S3 Primer sequences for the detection of the inserted CreER^{T2} sequence by PCR.

PCR product	Forward primer	Reverse primer
CreER^{T2} cassette	GGTGGTTCAGCTTACTTGCTAGAGC	TCAAATCAAGCTTCAAAATCCC
internal control	TAAACTGAGTCTTTGACAGGACATG	TCCCATGCACGCTAGGCAGGCTTTC

Development • Supplementary information

Supplementary references

Gouti, M., Delile, J., Stamataki, D., Wymeersch, F.J., Huang, Y., Kleinjung, J., Wilson, V., and Briscoe, J. (2017). A Gene Regulatory Network Balances Neural and Mesoderm Specification during Vertebrate Trunk Development. In Dev Cell (Elsevier Inc.), pp. 1-33.

Gouti, M., Tsakiridis, A., Wymeersch, F.J., Huang, Y., Kleinjung, J., Wilson, V., and Briscoe, J. (2014). In Vitro Generation of Neuromesodermal Progenitors Reveals Distinct Roles for Wnt Signalling in the Specification of Spinal Cord and Paraxial Mesoderm Identity. In PLoS Biol (Public Library of Science), pp. e1001937.

Koch, F., Scholze, M., Wittler, L., Schifferl, D., Sudheer, S., Grote, P., Timmermann, B., Macura, K., and Herrmann, B.G. (2017). Antagonistic Activities of Sox2 and Brachyury Control the Fate Choice of Neuro-Mesodermal Progenitors. Dev Cell *42*, 514-526.e517.

Simon, R., and Lufkin, T. (2003). Postnatal lethality in mice lacking the Sax2 homeobox gene homologous to Drosophila S59/slouch: evidence for positive and negative autoregulation. Mol Cell Biol *23*, 9046-9060.
